# Effect of Varying RuO_2_ (100) Thin-Film Stoichiometry on the Activity-Stability Compromise in the Oxygen Evolution Reaction: Pathway to RuO_2_ Degradation

**DOI:** 10.1021/acsomega.6c07471

**Published:** 2026-09-19

**Authors:** Ikenna Chris-Okoro, Ghanashyam Gyawali, Sheilah Cherono, Mengxin Liu, Wisdom Akande, Swapnil Nalawade, Brianna Barbee, Shobha Mantripragada, Shyam Aravamudhan, Veluchamy Palaniappagounder, Dhananjay Kumar

**Affiliations:** † Department of Mechanical Engineering, 3616 North Carolina A&T State University, Greensboro, North Carolina 27411, United States; ‡ Joint School of Nanoscience and Nanoengineering, 3616 North Carolina A&T State University, Greensboro, North Carolina 27401, United States

## Abstract

This study investigates the electrochemical properties of ruthenium dioxide RuO_2_ (100) thin films to understand the effect of varying oxygen deposition ambience on the activity-stability compromise and the degradation pathways consisting of the Lattice Oxygen Mechanism (LOM) and Adsorption Evolution Mechanism (AEM). Electrochemical evaluation of the RuO_2_ films in the oxygen evolution reaction (OER) in both alkaline and acidic media shows a lower overpotential for less oxygenated films than for more oxygenated films. Stability analysis reveals that, under alkaline conditions, the less oxygenated RuO_2_ thin-film samples experience a ∼16.5% reduction in current density, while the more oxygenated films experience a ∼9% reduction after 3 h. In acidic conditions, a higher current reduction of ∼21% was observed for the less oxygenated samples, whereas an improved current output of up to 17% was observed for the more oxygenated samples. The Ru dissolution trend for less oxygenated and more oxygenated films showed more than an order of magnitude variation under alkaline conditions and less than 1 order of magnitude under acidic conditions. Additionally, Ru metal dissolution increased by an average of 2 orders of magnitude under acidic conditions compared to alkaline conditions. Faradaic efficiency (FE) was considerably low for the more oxygenated films under both acidic and alkaline conditions. Based on the observed high current density, low overpotential, and high FE, we hypothesize that while LOM is operative in less oxygenated samples, AEM is operative in more oxygenated samples.

## Graphical abstract

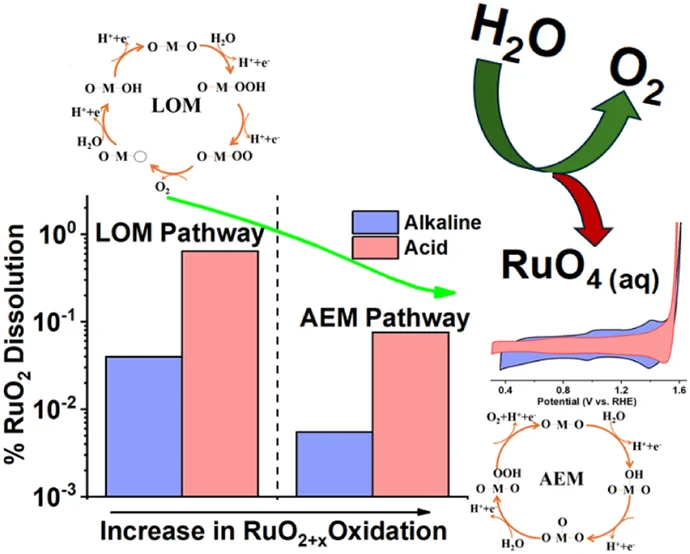

## 1 Introduction

Water oxidation catalysis, especially the Oxygen Evolution Reactions (OER), an anodic half-reaction, is essential for energy generation, materials science, corrosion science, environmental chemistry, and other sustainable technologies, including photoelectrochemical (PEC) devices,^1,2^ regenerative fuel cells,^3,4^ rechargeable batteries,^5^ electrochemical conversion of N_2_ − ammonia,^6–9^ CO and CO_2_ − hydrocarbons, alcohols, and phenols.^10–12^ However, a major challenge, especially for OER, is the harsh operational conditions and slow kinetics due to the 4-electron, 4-proton transfer to satisfy the reaction: 2 H_2_O _(aq)_ → O_2 (g)_ + 4 H^+^_(aq)_ + 4 e^−^ (E°_OER_ = 1.23 V vs RHE). This leaves these technologies with significant energy losses and temporal degradation of catalysts.^13,14^ Among the harsh operational conditions (which are more pronounced in acidic than in alkaline environments), and the limited precious-metal oxide catalysts that can maintain their stability at U ≥ 1.6 V vs RHE, RuO_2_ and IrO_2_ stand out. IrO_2_ is generally considered the best catalyst within the OER activity-stability compromise, although RuO_2_ is the more active electrode material, making its long-term stability a focus of several researchers.^15–22^

One of the major causes of OER instability in RuO_2_ is the overoxidation of Ru into a soluble, volatile, and high-valence (>4) species such as perruthenates or ruthenium tetroxide (RuO_4_),^15,23^ which is followed by the formation of oxygen vacancies (O_V_) during OER, leading initially to a breakdown in the crystalline structure of the electrode material and then the observed instability.^24,25^ In this light, researchers have adopted several stabilization techniques, primarily of which are the tuning of the electrode electronic structure vis-à-vis postgrowth treatment, vacancy engineering, crystal growth/orientation engineering, grain-dimensional control, anion doping, and cation doping so that the oxygen vacancy exceeds the 1.23 eV free energy of the redox couple H_2_O−O_2_.^4,22,26–35^

Among the various orientations of RuO_2_, the (110) orientation is the most studied due to its long-term stability under OER-relevant conditions; however, the (100) orientation is also of interest in the literature for its similar activity-stability properties.^36–38^ On the RuO_2_ (100) surface, there is evidence of RuO_4_ formation.^39,40^ Stoichiometric RuO_2_ (100) is (Figure 1a) usually formed at low oxidation temperatures with the presence of bridging O atoms (O_br_) directly bonded to coordinated unsaturated (cus) Ru_5f_ atoms (Ru_5f_ 5-fold). For charge neutrality at the RuO_2_ (100) surface, the densities of undercoordinated O_br_ and Ru_5f_ sites are similar.^41^ Upon surface reconstruction from the stoichiometric truncated RuO_2_ (100)-(1 × 1) because of overoxidation (Figure 1b), there is a reported loss of catalytic activity.^39–41^ This overoxidation increasingly results in the formation of the Ru_4f_ surface species (Figure 1c), a precursor to the volatile RuO_4_ and, eventually, to dissolution. From previous studies on RuO_2_ (110), it is clear that OH* adsorption is primarily due to dehydrogenation on the O_br_ sites, while conversion of OH* to O* is mainly due to dehydrogenation on the oxygen cus (O_cus_) sites.^21,42,43^ Contrasting the RuO_2_ (110) surface structure with RuO_2_ (100) reveals that, while RuO_2_ (100) stoichiometrically should have only bridge oxygen atoms, upon further oxidation, the absorbed oxygen (O_ot_) takes a cus position until the formation of the Ru_4f_ species.

**Figure 1:**
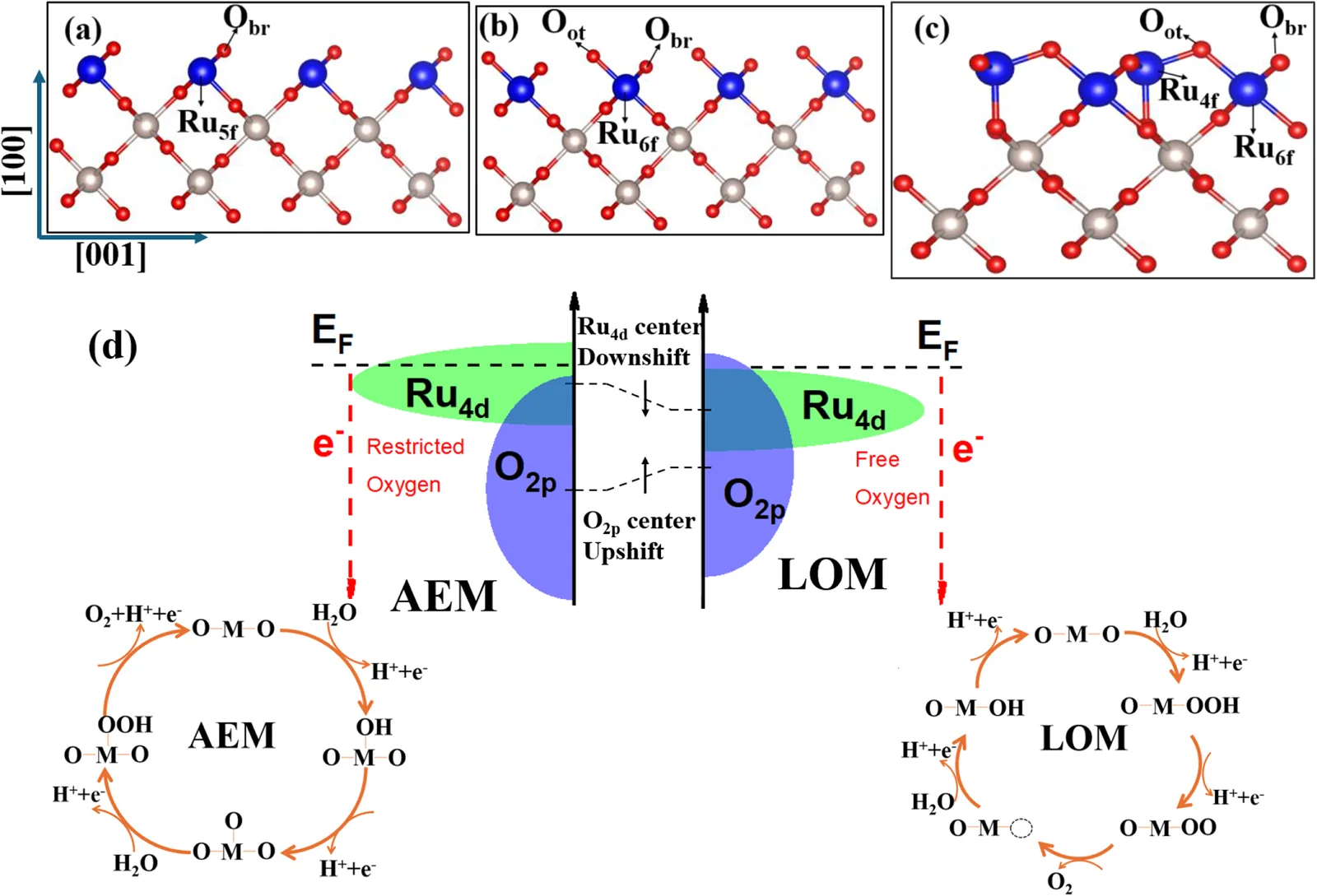
(a) Stoichiometric RuO_2_ (100) surface with the characteristic Ru_5f_ bonded to the bridging oxygen (O_br_), (b) RuO_2_ (100) surfaces with adsorbed oxygen in the cus position (O_ot_) with the characteristic Ru_6f_−O_ot_ pair, (c) hyper-stoichiometric RuO_2_ (100) surface where the Ru_6f_−O_ot_ pair undergoes reconstructions to the 4-fold coordinated Ru_4f_, adapted from the work of with their permission from Hess et al. (2022),^39^ Copyright 2022 American Chemical Society; and (d) schematic diagram of the AEM and LOM alongside illustrating their respective position of the O 2p and metal 4d bands, adapted from the work of and with permission from Kim et al. (2025),^48^ Copyright 2025 American Chemical Society. The LOM is favored due to the high covalency of the bond between Ru−O and the proximity of the O 2p band center to the Fermi level, while the AEM is the opposite case. The transition from AEM to LOM involves a reduction in the band gap, as indicated by the upshift of the O 2p and Ru 4d band centers.

In the OER, there are 2 major mechanisms for adsorption/conversion of intermediates and oxygen evolution: (a) Adsorption Evolution Mechanism (AEM) and (b) Lattice Oxygen Mechanism (LOM). In the Adsorption Evolution Mechanism (AEM), the correlation between the activity and catalyst structure is presented in the conventional 4-step, proton−electron transfer process for H_2_O*→OH*→O*→OOH*→O_2_ in OER, usually at the metal site without necessarily involving the lattice oxygen. The Lattice Oxygen Mechanism (LOM), favored by higher metal−oxygen covalent bonds, circumvents the limitations of AEM and bypasses the OOH* step, leading to a reduction in the catalyst overpotential.^44^ There is a well-defined structure−property relationship in transition metal oxides (TMOs) for OER relating to the oxygen 2p band center;^24,25,45,46^ and, in theory (ref Figure 1d), the AEM. The AEM presents a restriction of the lattice oxygen by the cation species, unlike in the LOM, where, due to orbital hybridization between the metal d band and the O 2p band, there is freedom for the lattice oxygen, enabling its participation in the OER reactions and the coupling to form the O−O bonding. During OER reactions, the AEM mechanism forms metal−oxygen bonds, and the LOM mechanism forms direct O−O bonds without necessarily forming metal−oxygen bonds. In earlier studies, the incorporation of oxygen vacancies (O_V_) into metal oxides has been shown to enable a transition from the inefficient AEM to the more efficient LOM for OER.^35,47^ However, the presence of these O_V_ could induce lattice distortion, affect the crystal structure, and serve as a precursor to the formation of the Ru_4f_ species and the eventual volatile RuO_4_. Ultimately, enhancing activity by increasing the LOM mechanism (vis-à-vis O_V_) compromises the catalyst’s long-term stability.

In this study, an attempt is made to provide an understanding of this activity-stability compromise in RuO_2_(100) by examining the effect of vacancies across a wide range of stoichiometries. Focusing on the transition from the AEM to the LOM and its effect on the rate of depletion of the RuO_2_(100) catalyst.

## 2 Methodology

Ruthenium oxide thin films from 99.99% high-purity RuO_2_ target were grown on c-plane sapphire (0001) substrates (10 × 10 × 0.5 mm^3^) by using a pulsed laser deposition (PLD) technique in varying oxygen ambience conditions, i.e., 2.9 × 10^−3^ mTorr O_2_, 5 mTorr O_2_, 25 mTorr O_2_, 50 mTorr O_2_, 75 mTorr O_2_, 100 mTorr O_2_. A Krypton Fluoride-KrF Laser PLD (Coherent Complex Pro, wavelength 248 nm, pulse duration 25 ns) chamber was used. Before deposition, the chamber was pumped down to a base pressure of ∼1.5 × 10^−6^ Torr before deposition commenced. The target-substrate distance was fixed at 52 mm, the laser frequency was 10 Hz, and the laser energy density was also kept at 2.1−2.3 J/cm.^2^ During deposition, the substrate was also maintained at 600 °C and postdeposition cooled in >50 Torr O_2_ (ref Table S1).

A fixed number of 5000 laser pulses was used at 10 Hz to maintain sample consistency. To verify the authenticity and reproducibility of the data presented in this work, the samples were deposited 3 times. The sapphire substrates were prepared by using the method described in our earlier publication.^49^ The unit lattice models were simulated using visualization for electronic and structural analysis (VESTA).^50^

The film−substrate orientation, thickness, and crystallinity were determined using an X-ray diffractometer (Rigaku SmartLab XRD) with a Cu Kα X-ray source (λ = 0.154 nm) with an applied voltage of 40 kV and 150 mA. Film electrical resistivity was measured using the Ossila T2001A standard 4-probe measurement. Film composition was measured using a Lab-based ThermoFisher Escalab Xi+ working with Al Kα monochromatic radiation (up to 1486.6 eV) under ultrahigh vacuum (UHV) conditions at base pressure ∼10^−9^ Torr with a source-to-analyzer angle of 54.7°. High-resolution X-ray photoelectron spectroscopy (XPS) spectra were recorded for the Ru 3d, Ru 3p, and O 1s core levels. O 1s core levels were fitted using the fit procedure with Shirley background subtraction with 100 and 50 eV pass energy for survey and high-resolution scans, respectively, for a reasonable signal-to-noise ratio. The reproducibility and accuracy of the fitting analysis were only accepted if the normalized residual chi-square (χ^2^) variation was <9 units. ThermoFisher Avantage software was used to fit the XPS data with symmetric line shapes (L/G − 30/70), comprising 30% Lorentzian and 70% Gaussian, and with some asymmetric line shapes formed by interactions created during the photoemission process by holes and mobile conduction electrons. The inelastic mean free path (IMFP) at 1486.6 eV was calculated to ∼5.02−6.43 nm (based on the 3 λ probing depth, λ is X-ray wavelength) for RuO_2_ and its higher-binding species (RuO_4_, etc.) measured for the O 1s with Incident Photon Energy of 530 eV based on Tanuma−Powell−Penn (TPP)-2 M equations using QUASES software and as described for NIST IMFP databases.^51–54^ As the C 1s falls under the Ru 3d, it was difficult to reference all high-resolution spectra to it, so an effort was made to use a freshly grown PLD gold film (Au 4f_7/2_ at 83.96 eV) for binding-energy referencing and spectral shifting. These XPS measurements were taken before and after electrochemical analysis in both acid and alkaline conditions. The WiTec alpha300R Confocal Raman Microscope, with a laser excitation wavelength of 532 nm (green visible light), was used to measure Raman Spectra. The surface morphology was also measured using atomic force microscopy (AFM; Asylum Research Jupiter XR) at various scan sizes.

The electrochemical properties of RuO_2_ thin films were measured using a three-electrode configuration employing an electrochemical workstation potentiostat (Bio-Logic, SP-300). Ag/AgCl, saturated with KCl, was used as the reference electrode, with the PLD-grown RuO_2_ thin films and a Pt coil wire serving as the working and counter electrodes, respectively. The three-electrode setup used 0.1 M HClO_4_ and KOH (99.99% purity, Sigma-Aldrich) as acidic and alkaline electrolytes, respectively. These measurements include cyclic voltammetry (CV), linear sweep voltammetry (LSV), electrochemical impedance spectroscopy (EIS), and chronoamperometry (CA). LSV was recorded at a scan rate of 10 mV s^−1^ over a potential range of 0.30 to 1.81 V vs RHE. The CV was recorded at scan rates of 20−200 mV s^−1^, with a step increase of 20 mV s^−1^, over potential ranges of 0.76−0.86 V vs RHE and 0.30−1.61 V vs RHE. The EIS was recorded by applying an oscillatory voltage with an amplitude of 10 mV over a frequency range from 200 kHz to 1 Hz. Nyquist plot analysis was performed at various voltages vs RHE: 1.51, 1.56, 1.61, 1.66, and 1.71. The CA analysis was recorded stepwise at potentials of 1.81, 1.76, 1.71, and 1.66 V vs RHE over 3 h. Afterward, it was also measured at 1.9 V vs RHE for 12 h. The OER activity of the RuO_2_ thin films was evaluated by comparing the overpotential required to achieve a current density of 1 mA cm^−2^. 10 mA cm^−2^ is the most used benchmark for OER catalysts as it roughly matches the hydrogen and oxygen production rate of a 12.3% efficient solar-to-hydrogen conversion device under one sun of sunlight.^1,2^ In the present work, we report the overpotential at 1 mA cm^−2^ because a current density of 10 mA cm^−2^ is rarely achieved in thin-film samples. This is consistent with results in the literature on epitaxial thin-film RuO_2_ electrocatalysts,^36–38^ which report overpotentials at 0.1 mA cm^−2^. Moreover, using 1 mA cm^−2^ enables reliable comparisons among films with different compositions while minimizing the influence of mass-transport limitations, catalyst-loading variations, and possible degradation effects at higher current densities.

The potential obtained using the Ag/AgCl reference electrode was converted to a reversible hydrogen electrode (RHE) by *E*_RHE_ = *E*_Ag/AgCl_ + 0.0591 × pH + *E°*_Ag/AgCl_, where *E*_RHE_ = potential vs RHE, *E*_Ag/AgCl_ = actual potential vs Ag/AgCl, *E*°_Ag/AgCl_ = standard electrode potential of Ag/AgCl electrode (0.197 V vs normal hydrogen electrode—NHE at room temperature), and pH = 13 for 0.1 M KOH, pH = 1 for 0.1 M HClO_4_. The overpotential (η) of electrocatalysts at 1 mA cm^−2^ in this case, for the OER, was calculated using η_OER_ = (*E*_RHE_ − 1.23) V vs RHE, where *E* signifies the electrochemical potential vs RHE.

The dissolution of RuO_2_ thin films was quantified by inductively coupled plasma mass spectrometry (ICP-MS). The concentration of dissolved Ru ions in the electrolyte was analyzed using an Agilent 7900 quadrupole ICP-MS, with calibration performed using a 1000 mg L^−1^ Ruthenium ICP standard solution prepared from RuCl_3_ in 7% hydrochloric acid (HCl). The calibration curve was prepared over the 0.1−100 ppb Ru range, and the electrolyte solutions were measured after 12 h chronoamperometry at 1.9 V vs RHE. Although longer-term stability measurements are useful, extended tests have been reported to introduce uncertainty and reproducibility problems due to possible film-thickness gradients that lead to local resistivity variations, substrate exposure, redeposition of dissolved Ru species, etc.^55–57^ Therefore, the samples were initially cycled for 4 h, followed by rinsing to stabilize the surface and remove weakly adsorbed surface species. The final Ru concentration was obtained from the ICP-MS calibration curve and reported in parts per billion (ppb) and nanograms per milliliter (ngmL^−1^), where 1 ppb is approximately equivalent to 1 ngmL^−1^ for dilute aqueous electrolyte solutions. The reliability of these measurements was assessed by comparing the measured Ru dissolution values with reported literature trends for RuO_2_ instability and Ru dissolution under oxygen-evolution conditions. Further explanation is offered in the supplementary section.

## 3 Results and Discussion

The XRD analysis recorded from RuO_2_ thin films grown on *c*-plane single-crystal sapphire (Al_2_O_3_) substrates shows characteristic peaks of a tetragonal RuO_2_ crystal structure with a first harmonic (200) peak between 39.30° and 40.51° (a = b = 4.58−4.45 Å and c = 3.11 Å) and a second harmonic (400) peak between 84.55° and 87.63° for films deposited between 5 and 100 mTorr O_2_ (ref Table S2). This is explained by variations in stoichiometry (substoichiometric, stoichiometric, hyper-stoichiometric) from the theoretical 2θ positions for RuO_2_ (200) at 40.227° and (400) at 86.907°. As a standard sample grown in vacuum (0.0029 mTorr O_2_) shows the Ru (002) peaks at 42.15° and 92.10°, and a weak RuO_2_ peak (inset of Figure 2a). A high level of crystallinity was recorded for films with full-width at half maximum (fwhm) 0.312°−0.055° from the Omega Rocking Curves (ORCs) with minima at films with the best stoichiometry −50 mTorr O_2_ (Figure 2b, ref Table S1 and Figure S1). However, the vacuum sample recorded a larger fwhm at 1.033°. The film thickness, surface roughness, and density estimated are also presented in Table S1.

**Figure 2:**
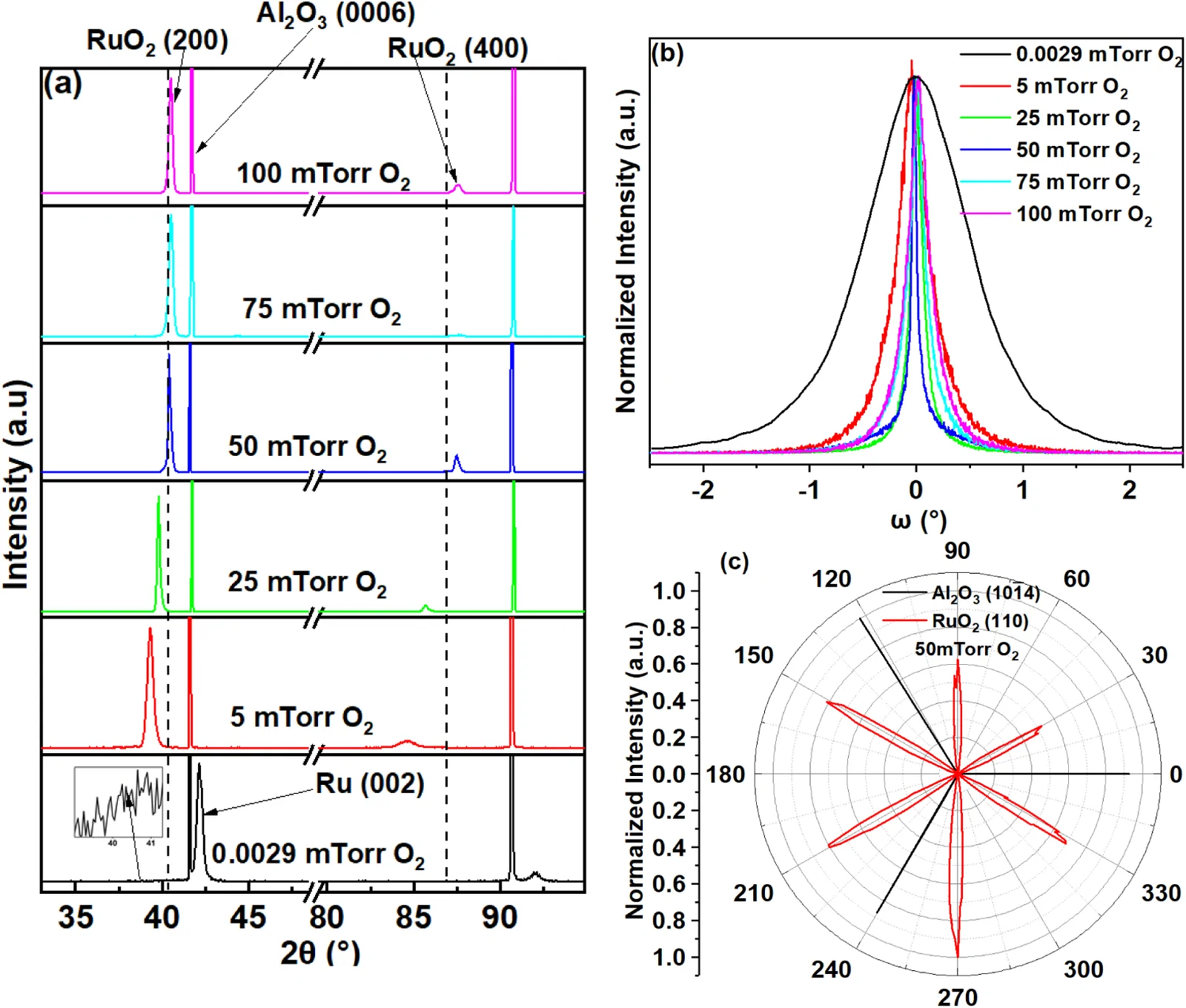
(a) X-ray diffraction patterns of the various RuO_2_ thin films deposited at varying oxygen ambience, (b) Omega rocking curves of the thin films as seen in (a), and (c) φ-Scan of the RuO_2_ 50 mTorr O_2_ film showing a 30° rotation of the (110) film plane with respect to the (101̅4) plane of the substrate.

The phi-scan measurement (Figure 2c) was taken to verify the in-plane epitaxial relationship between the film and substrate, with the rotation axis perpendicular to the film surface observed to be (100)_RuO2_//(0001)_Al2O3_ and [010]_RuO2_ // [101̅0]_Al2O3_. The observed reflections are in the RuO_2_(110) and Al_2_O_3_(101̅4) planes.

The atomistic renderings were developed using VESTA^50^ from the Materials Project, mp-1143 and mp-825 (Figure S1 c−f). These reflections from the RuO_2_ film are spaced at approximately 60° in the φ-scan (a 6-fold symmetry, C6−2D repeat pattern), and the reflections of the Al_2_O_3_ substrate are spaced at 120° in the φ-scan (a 3-fold symmetry, C3-triangular 2D repeat pattern). From the φ-scan, a ∼30° rotation of the film plane with respect to the substrate peak plane is observed. This result indicated the formation of triple domain structures (at 120° in phi and at ±30° from the sapphire (101̅4) peaks) in the films along the triangular axes of the substrate, with the in-plane epitaxial relationships RuO_2_[010]//α-Al_2_O_3_[101̅0], [1̅010], and [01̅10].^58–60^ The 30° rotation can be explicitly described using 2D unit-cell structures, where both the RuO_2_ film and the Al_2_O_3_ substrate are assumed to be oxygen-terminated. The 2D lattice parameters of Al_2_O_3_ are 4.81 Å, while those of RuO_2_ are 3.11 Å and 4.48 Å. The mismatch produced by this rotation is much less than the 7% required for conventional epitaxial film growth to take place (precisely about 5.75% in this case). This can be described as the RuO_2_ layer nucleating and straining at the interface with the Al_2_O_3_ substrate due to the positioning and rotation of oxygen atoms in the RuO_2_ film relative to the oxygen atoms in the Al_2_O_3_ substrate, leading to energetically favorable growth via domain epitaxy and cation−anion−cation bonding.^61–63^

To understand the relationship between structural information and surface chemical composition, Lab-based XPS was performed under ultrahigh vacuum (UHV) at room temperature (RT). The high-resolution XPS core-level spectra of Ru 3d, Ru 3p, and O 1s were analyzed. Due to spin−orbital coupling, a peak doublet is observed in the Ru 3d and Ru 3p XPS spectra (ref Figure 3 a−b), with spin splitting for all species kept within the range of 4.17 ± 0.1 eV and 22.20 ± 0.3 eV, respectively.^57,64–69^ From the Ru 3d spectra, a species related to the Ru^4+^-RuO_2_ (Lower Binding Energy − LBE RuO_2_) was observed at 280.675 ± 0.025 eV, and another related to the Ru^4+δx^- RuO_2+Δx_ (Higher Binding Energy − HBE RuO_2_) was observed at 281.785 ± 0.105 eV. This higher binding energy RuO_2_, though not well defined in the literature, is related to the formation of species that could include volatile RuO_3_, perruthenates, or ruthenium tetroxide (RuO_4_). At 283.085 ± 0.185 eV, a satellite peak was observed as a result of the shake-up phenomena common in transition-metal oxides. While there is debate in the literature on the viability of a stable phase of RuO_2+x_ and its proximity to the satellite and other unscreened final-state features,^70^ they do not account for the dissolution mechanism of RuO_2_ and its tendency toward overoxidation through the restructuring of Ru_5*f*/6f_ into Ru_4f_. As the Ru 3d and C 1s spectra overlap, we used the Au 4f (Figure S2) for binding-energy correction so that the quantification of surface carbon and its contribution to electrochemical stability can be reported with high confidence. From the C 1s peak, C−C (Adventitious) deconvoluted at 284.84 eV, and other carboxyl groups were subsequently deconvoluted (ref Tables S3−S4). As there was no peak feature below 280 eV, it can confidently be concluded that, for all samples at the surface, regardless of varying oxygen ambiance, there is no/negligible amount of Ru metal species. From the Ru 3p spectra, the lower binding energy RuO_2_ and higher binding energy RuO_2_ are also deconvoluted. The core-level O 1s spectra acquired for each sample have been deconvoluted into species from the lower binding energy RuO_2_, higher binding energy RuO_2_, a species representing the adsorbed hydroxyl/carbonate and chemisorbed O species a representative of a variety of species such as C−OH, C−O, C=O, adsorbed water, and other atmospheric vapor oxides which explains the large fwhm (ref Table S5). For transparency, the raw data, normalized and shifted, are illustrated in Figure S3. These spectra are visually similar, indicating no significant change in the electronic structure of the various thin films, most likely due to the <50 Torr postdeposition ambiance.

**Figure 3:**
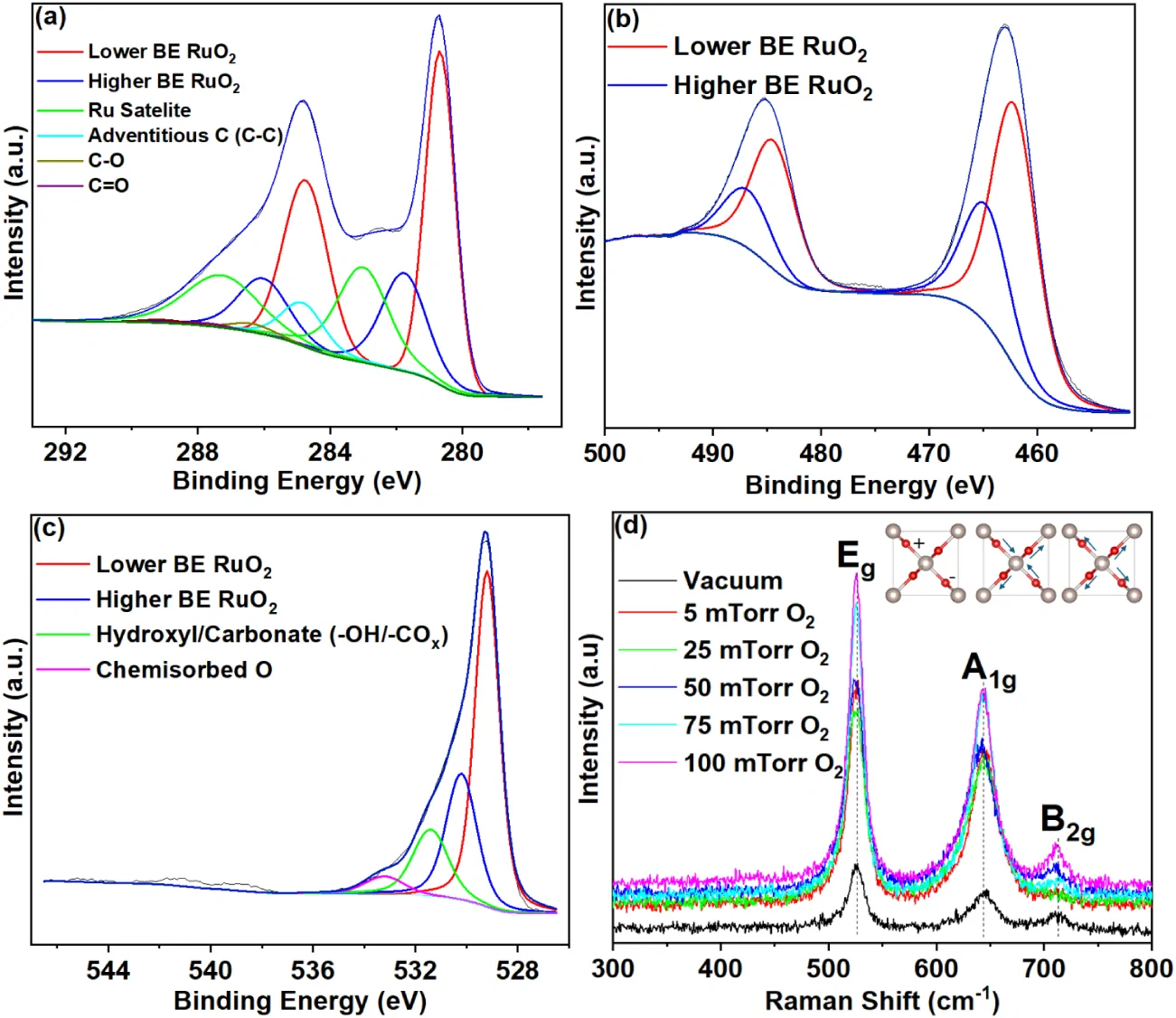
XPS high-resolution spectra at (a) Ru 3d core level, (b) Ru 3p core level, (c) O 1s core level, and (d) Raman spectra from 532 nm wavelength laser excitation from the various RuO_2_ thin films deposited in varying oxygen ambience.

From the analysis of the data, an inverse exponential relationship between the ratio of lower binding energy RuO_2_ to the higher binding energy RuO_2_ (Figures S4−S5) shows an increase in the high valence (>4) Ru with some degree of uncertainty, some of which could be volatile with increasing oxygen ambience of the deposition. The correctness of this fitting approach is underscored by the precision of the interval between the 3p_5/2_ and 3p_3/2_-doublet peaks, the similarity of the fwhm with literature, and the agreement between the species ratios in the Ru 3d and those of the Ru 3p and O 1s (ref Tables S3−S5). Similarly, the constancy of the ratio of ruthenium to oxygen across all samples (ref Figure S6) indicates an insignificant contribution of carbon in the electrochemistry of these films. This is always a concern, as the carbon concentration on surfaces has been shown to enhance electrochemical activity. While the depth of the electrochemical reaction, more specifically the active layer, varies across thin-film surfaces^71^ (typically ranging from 1 to 50 nm), this constancy in the surface-depth ratio ensures uniform charge transport. That degradation was more systematic over time.

To further understand structural ordering and phase purity, Raman spectroscopy was performed on all samples. The Raman spectra (Figure 3d) show the characteristic vibrational modes of the rutile phase of tetragonal RuO_2_ at ∼526 cm^−1^, ∼623 cm^−1^, and ∼711 cm^−1^, the E_g_, A_1g_, and B_2g_ modes, a representative of epitaxial RuO_2_ (Figure 3d). The inset 2D illustration shows the symmetry of Raman-active modes at the point (zero wave vector), all of which involve only oxygen-atom displacements. The increase in peak intensity is also observed in higher-oxygen-deposition ambiance, resulting from elevated crystallinity and structural order, and improved anion doping in the various films.

The electrochemical activity and stability of the various deposited RuO_2_ thin films were explored using cyclic voltammetry (CV) to examine their redox behavior. The potential measurement range was maintained at 0.3 V−1.61 V vs RHE and was taken after 20-cycle scans were taken for stabilization (however, there were no significant spectral differences between the first and 20th) in both alkaline (0.1 M KOH, ref Figure 4a) and acidic media (0.1 M HClO_4_, ref Figure 4b, Figures S7−S10). For the various thin films, broad redox features are observed at ∼1 V and ∼1.4 V vs RHE in alkaline conditions, and ∼0.8 V, ∼1.2 V, and ∼1.5 V vs RHE in acidic conditions during the cycling scan. These characteristic steps for epitaxial RuO_2_ (100) represent the OH* intermediate (where’*’ is used to denote intermediates adsorbed to active sites), the conversion of OH* to the O* intermediate, and the conversion of O* to the OOH* intermediate, respectively, preceding OER onset. The presence of these distinct redox peaks in both conditions after several cycles indicates that RuO_2_ (100) can undergo reversible redox intermediate conversions/reactions, which are essential for studying OER mechanisms. In general, the shapes of the CV curves and the redox positions on the potential scale vary between the two electrolytes. The basic OER mechanisms vary depending on the source of oxygen atoms and the species generated, as observed from reaction kinetics and thermodynamics; in acidic conditions, water oxidation occurs, while in alkaline conditions, hydroxide oxidation occurs.^72–74^ However, in both conditions, the typical AEM pathway maintains 4 proton−electron transfer steps, with adsorption and conversion of oxygen-related intermediates (OH*, O*, and OOH*), as shown in eqs 1 and 2 below.

1 $${2H}_{2}O^{*}\to {\mathrm{OH}}^{*}{\;+\;H}_{2}O\;+\;\left(H^{+}{\;+\;e}^{-}\right)\to O^{*}{\;+\;H}_{2}O\;+\;\left({2H}^{+}{\;+\;2e}^{‐}\right)\to {\mathrm{OOH}}^{*}\;+\;\left({3H}^{+}{\;+\;3e}^{-}\right)\to O_{2}\;+\;\left({4H}^{+}{\;+\;4e}^{-}\right)\;\mathrm{in acid environment}$$

2 $${\mathrm{4OH}}^{-}\to {\mathrm{OH}}^{*}{\;+\;\mathrm{3OH}}^{-}{\;+\;e}^{-}\to O^{*}{\;+\;\mathrm{2OH}}^{-}{\;+\;H}_{2}{O\;+\;2e}^{‐}\to {\mathrm{OOH}}^{*}{\;+\;\mathrm{OH}}^{-}{\;+\;H}_{2}{O\;+\;3e}^{‐}\to O_{2}{\;+\;2H}_{2}{O\;+\;4e}^{-}\;\mathrm{in alkaline environment}$$

**Figure 4:**
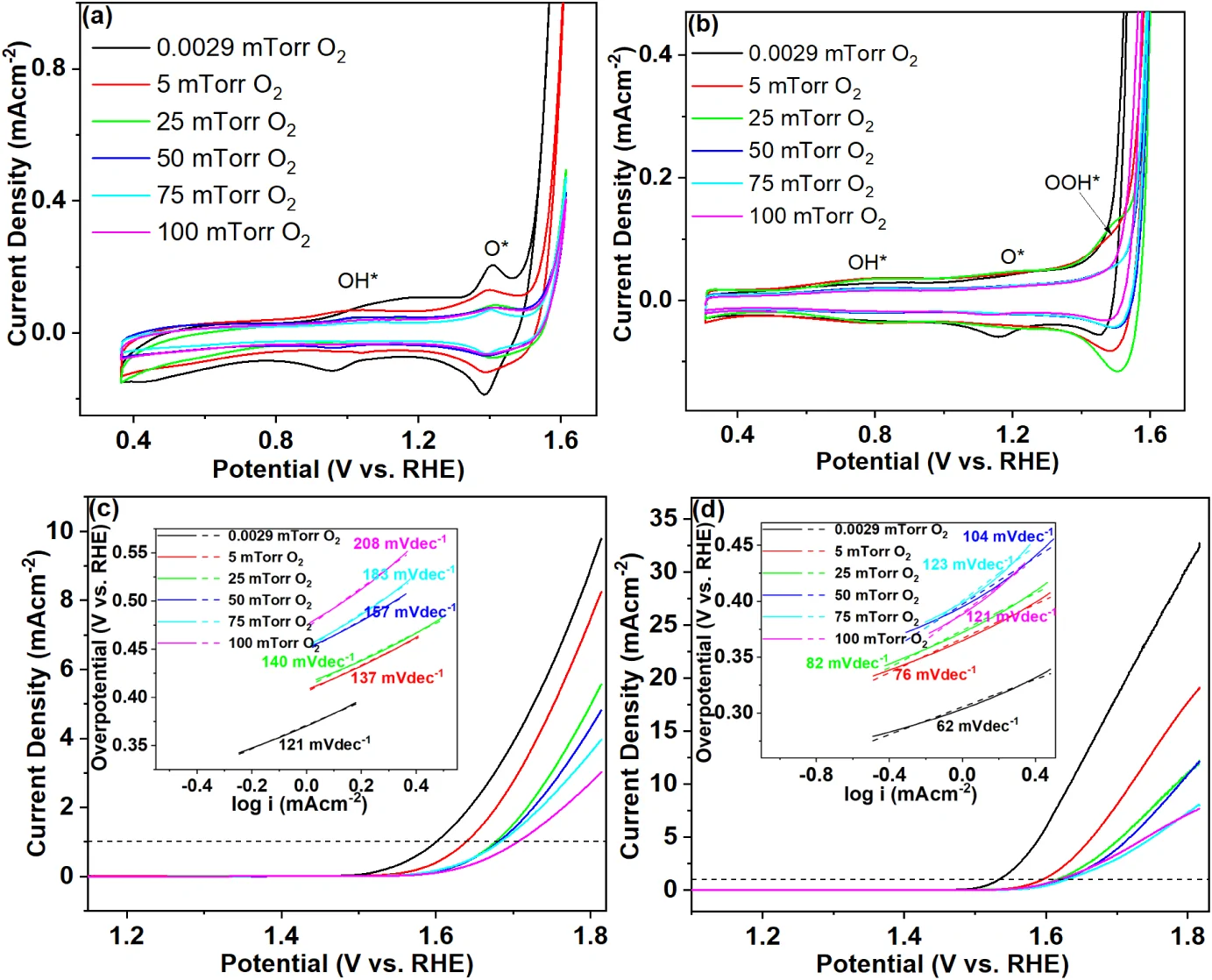
CV curves recorded at 200 mVs^−1^ for the various RuO_2_ films: (a) 0.1 M KOH, (b) 0.1 M HClO_4_. Linear-sweep voltammetry curves recorded at 10 mVs^−1^ for the various RuO_2_ films, with the inset showing the Tafel slope for (c) 0.1 M KOH and (d) 0.1 M HClO_4_.

In contrast, the LOM pathway initially mirrors the AEM; however, it bypasses the OOH* intermediate formation, a process in which the adsorbed O* species bond with the lattice oxygen within the RuO_2_ catalyst structure, resulting in O−O bond formation and O_2_ evolution. This evolution creates additional surface vacancy sites, which, upon contact with H_2_O molecules, form an OH* intermediate, and the cycle continues. As shown in previous studies, a substantial number of vacancies can shift the pathway significantly from LOM to AEM. These defects (vacancies) tune the d-band center of Ru relative to the Fermi level, effectively reducing the adsorption energy (ΔG) of intermediate species and facilitating cohesive interactions between active sites and adsorbed intermediates. Similarly, the presence of these oxygen vacancies increases the O 2p-band center from −4.57 eV in pristine RuO_2_ to −3.03 eV in the CoRuO_*x*_.^75^ It reduces the formation energy of oxygen vacancies (ΔG_OV_) from 5.68 eV in RuO_2_ and 5.29 eV in Cr_0.5_Ru_0.5_O_2_.^76^

From the results presented in the CV analysis, we observed mechanistic variations across the various films. For instance, in the case of RuO_2_ films deposited in high vacuum conditions (0.0029 mTorr = 2.9 × 10^−6^ Torr), we have observed that the O* redox peak in the alkaline medium is well-defined (higher current density) and sharper (lower fwhm) than the peaks in other samples. This difference in the peak feature of the O* redox peak, along with the fact that it begins to evolve oxygen at low potential, shows a tendency toward the ideal Nernstian limit (90.6/n mV at 25 °C for a surface-bound species), indicating a highly reversible process without the more sluggish electron-transfer kinetics. This occurs alongside a hypothetical lowering of the overpotential barrier for OOH* formation (the rate-limiting step) and possibly a high degree of metal−oxygen covalency with respect to the band structure, which could make these films susceptible to overoxidation and Ru dissolution. Also, in acid, at 0.0029 mTorr O_2_ we observed that oxygen evolution occurred in the same potential region where OOH* formation was observed for other samples, which could partially confirm the earlier hypothesis (transition from AEM to LOM). Although the 50 mTorr O_2_ sample exhibits the highest crystallinity, catalytic stability is not to be determined solely by crystallinity. The oxygen stoichiometry, defect concentration, Ru−O bonding environment, and surface reconstruction behavior under OER conditions also play critical roles. Oxygen-deficient samples contain more oxygen vacancies and enhanced lattice-oxygen mobility, which can promote OER kinetics and improve activity but may also accelerate oxygen loss, Ru dissolution, and structural degradation, reducing stability. The different activity trends of the 100 mTorr sample in acidic and alkaline electrolytes are attributed to the distinct surface reaction environments and reconstruction processes. The balance between defect chemistry, Ru oxidation state, and electrolyte-dependent surface intermediates determines the catalytic response.

Similarly, the electrochemical activity was further evaluated by linear sweep voltammetry (LSV) in both alkaline (ref Figure 4c) and acidic (ref Figure 4d) conditions. From the polarization curves, an overpotential was recorded at 1 mA cm^−2^ to be between 370 and 476 mV in alkaline conditions and 308−399 mV in acidic conditions (ref Figure S11). The reaction kinetics were also represented by the fitting of the Tafel slope, shown in the insets of Figure 4c for alkaline and Figure 4d for acidic conditions. The slope ranges from 121−208 mVdec^−1^ in alkaline and 62−123 mVdec^−1^ in acidic conditions, implying better OER kinetics in acidic than in alkaline conditions. These results emphasize improved electron transfer for the OER, especially for samples deposited in a low-oxygen ambiance, with acidic conditions also providing an avenue for reduced overpotential.

To further understand the stability of the various RuO_2_ (100) thin films, CA measurements were undertaken (Figure 5) by applying varying potentials of 1.81, 1.76, 1.71, and 1.66 V vs RHE in a stepwise fashion above the potentials of the redox couples previously observed, and the OER onset in both alkaline and acidic conditions. This was done specifically to observe the reversibility of the samples (current retention) under corrosive operational conditions, as most industrial OER electrocatalysts are typically operated between 1.6 and 2.0 V vs RHE. From the results in 0.1 M KOH (Figure 5a), a ∼16.5% reduction in current density is observed after 3 h for the 0.0029 mTorr O_2_ sample, but this is improved to ∼9% for the 100 mTorr O_2_ samples over the same time. Similarly, LSV results were obtained after the CA test and compared with the initial LSV results (Figure 5c), as an increase in the overpotential of all samples was observed after the CA test in alkaline conditions, ranging from 8 mV to 43 mV at 1 mA cm^−2^ for the various films in increasing order of deposition ambiance.

**Figure 5:**
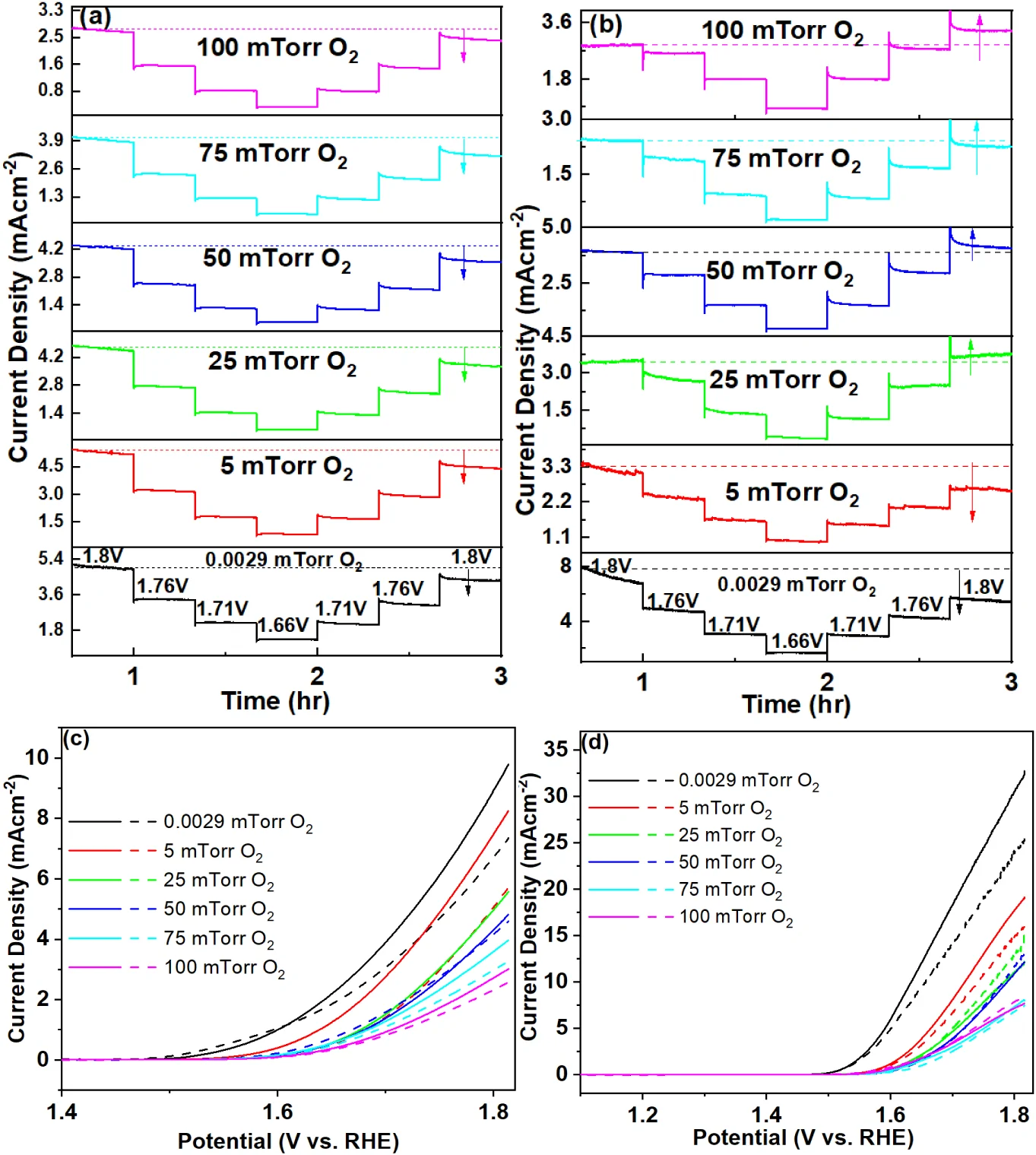
CA plots for various RuO_2_ films performed at different potentials for a period of 3 h. in (a) 0.1 M KOH and (b) 0.1 M HClO_4_, and LSV curves for the same films before (thick lines) and after the CA tests (dotted lines) in (c) 0.1 M KOH and (d) 0.1 M HClO_4_.

Interestingly, in 0.1 M HClO_4_ (Figure 5b), the 0.0029 mTorr O_2_ sample showed a higher current reduction of ∼21%. Still, for samples deposited under a higher oxygen atmosphere, current output improved after the 3 h cycle, up to a 17% improvement at 100 mTorr O_2_. This could be attributed to adsorption of the oxychloride group on the surface of the RuO2 films, which occurs during the initial potential variation from high to low during electrodeposition, leading to the formation of RuOClx, which has been reported to improve OER activity and stability.^77^ Comparing the change in activity from the pre- and post-LSV analysis (Figure 5d), it is observed that while the 0.0029 and 5 mTorr O_2_ samples show a reduction in activity, the other samples, aside from the 75 mTorr, show improvement in activity and lower overpotential.

Analyzing the Electrochemical Impedance Spectroscopy (EIS) data (Figure S12), the charge-transfer resistance (R_ct_), a function of electron transfer between the RuO_2_ and the electrolyte (an inverse indicator of corrosion rate), was measured before, during, and after OER onset. A downward trend in geometric progression was observed for all samples as the potential was increased from 1.51 to 1.71 V vs RHE, indicating that corrosion increased significantly over the 0.2 V range. Likewise, the Tafel plots (ref Figure S13, Tables S6−S7) used to study the susceptibility of RuO2 (100) to pitting corrosion show a steady trend of increasingly positive corrosion potential as the RuO_2_ samples increase in oxygen-deposition ambiance, peaking at the 50 mTorr O_2_ sample. This indicates a lower tendency toward corrosion and improved corrosion resistance as the samples transition from substoichiometric to stoichiometric states (ref Figure S1b). However, as oxygen pressure increases toward the hyperstoichiometric state, corrosion potential decreases. This creates a parabolic relationship between the stoichiometric state of RuO_2_(100) and its tendency toward corrosion resistance. This confirms that increasing the oxygen level of RuO_2_ above stoichiometry does not improve corrosion resistance, as reported in some papers. This trend is also confirmed by the corrosion current density (optimal: high E_corr_, and low I_corr_). In general, the absence of a hysteresis loop points to the nonexistence of pitting corrosion, although general corrosion could still be a major factor. This is important especially for the substoichiometric RuO_2_ samples, where the vacancies are created by the formation of the O−O bond and its eventual evolution is expected to lead to localized corrosion (pitting corrosion). Although the Electrical Double Layer Capacitance measured as a function of current and scan rate (ref Figure S14) shows generally lower double-layer capacitance in acidic electrolysis, which should indicate improved corrosion resistance.

Analyzing the surface of the various RuO_2_ films after electrochemical analysis, for the presence of metal that could indicate catalyst poisoning and the after-effect of electrolyte contamination from Ag, Pt, Fe, Cu, Ni, Co, Mn, etc.; as seen from the post-CA XPS survey, the chemical purity of the RuO_2_ (100) catalyst was largely retained with no noticeable contaminant adsorption (ref Figure S15). This is essential for reproducible results. From the Ru 3d core level (ref Figures S16−S17), no significant differences are noted at first glance, no real peak shift (binding energies), also no growing peak at the lower binding energy side (∼280.6 eV−283.5 eV), but some in the higher binding energy side (∼284.0 eV−286.5 eV), which can be initially ascribed to an increase in adventitious carbon. However, on further fitting, as seen in Figure 6a−c, an evolving trend is observed. In relation to the over oxidation of RuO_2_ (based on the area ratio) due to the harsh operational conditions of the OER, an inverse trend is noted in both acidic and alkaline electrolytes from the as-deposited, where the more oxidized samples seem to shed more HBE RuO_2_ than when compared to less oxidized ones that instead accumulate more on the surface. Since the rate of increase in HBE RuO_2_ is within the error interval or lower than the initial hypothesis, it can be assumed that the rate of over oxidation of RuO_2_ is generally low, or/and that the formation and dissolution of RuO_2+x_ is mostly an in situ process. A similar trend is also observed for the Ru 3p spectra, with a shoulder hump between ∼456 eV and ∼459 eV, and ∼480 eV and ∼475 eV, which was not deciphered during this project.

**Figure 6:**
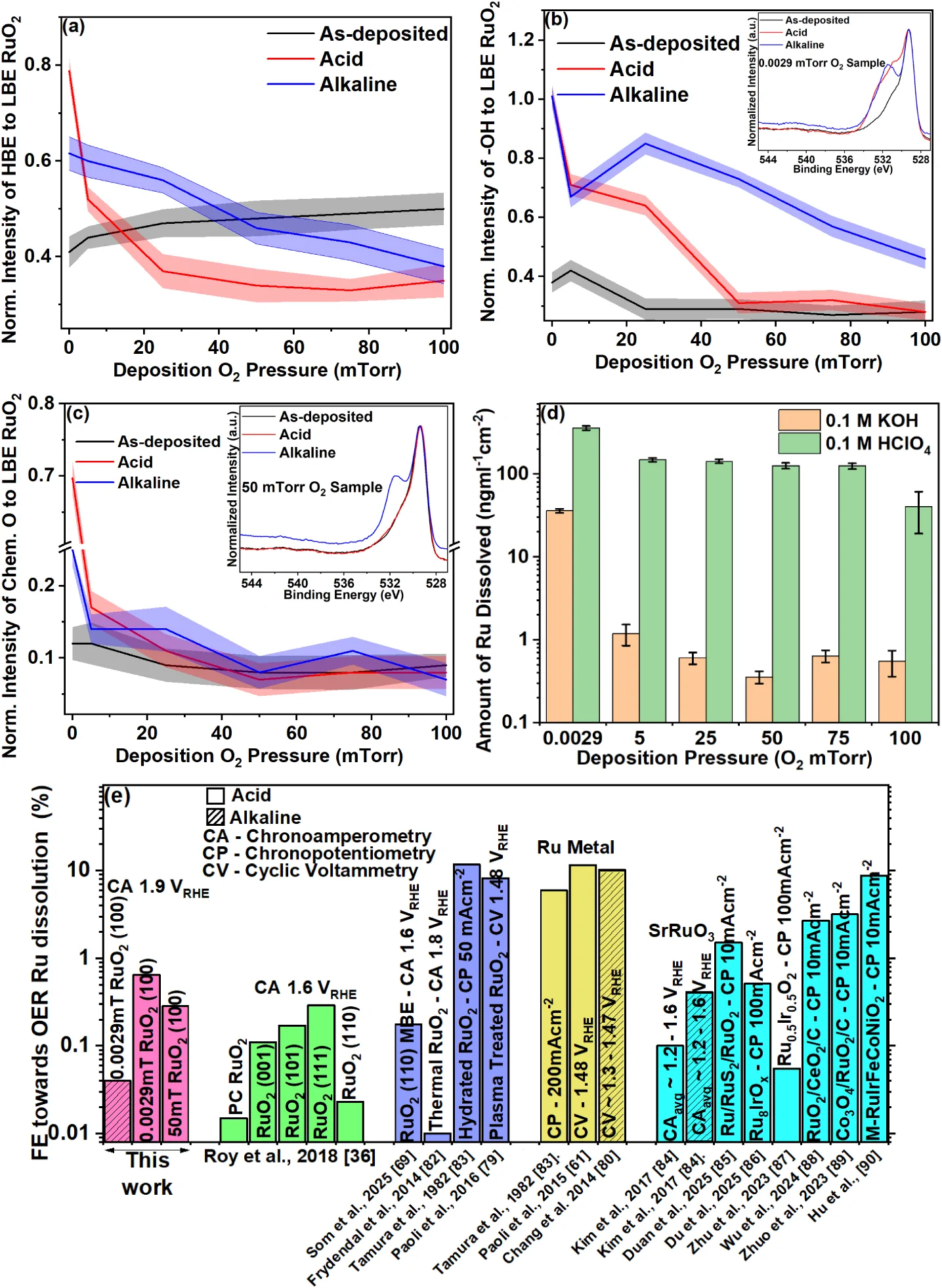
Normalized area ratios: (a) HBE RuO_2_, (b) adsorbed −OH (which could contain some carboxyl group- CO*_x_*) with inset the O 1s XPS spectra for as-deposited, after 0.1 M KOH and 0.1 M HClO_4_ analysis for 0.0029 mTorr and 50 mTorr O_2_ sample, respectively, (c) chemisorbed O (the majority of which is adsorbed water-H_2_O), (d) the amount of dissolved Ru metal from the various RuO_2_ thin film electrodes during a potential scan from 1.96 V vs RHE, higher than the OER onset in both 0.1 M KOH (alkaline) and 0.1 M HClO_4_ (acid), and (e) the Faradaic efficiency of dissolution for various RuO_2_ thin film electrodes in both 0.1 M KOH and 0.1 M HClO_4_.

Interestingly, major changes were observed in the O 1s core-level analysis in Figure 6 b−c, with the inset images showing spectral modification due to electrochemical polarization in the region between ∼530 eV and ∼535 eV. From the deconvolution, the spectra relating to the bulk oxides (HBE and LBE RuO_2_) are separated from other adsorbates (chemisorbed O group, which is composed of a good amount of adsorbed H_2_O and −OH group, which could contain some CO_*x*_ and OCl_*x*_ in the case of postacid test). Most spectral changes arise from the adsorption and dissolution of water on electrocatalyst surfaces, which occurs when water molecules (acidic electrolyte) and/or hydroxyl groups (alkaline electrolyte) are adsorbed, usually at unsaturated metal sites (cus-sites) and eventually at bridge sites. At higher oxygen levels (50−100 mTorr O_2_ samples), these adsorptions are noted not to vary largely from the initial state, which, with reference to the structure of the RuO_2_ (100) (Figure 1b), are due to the saturation of adsorbed oxygen in the cus position (O_ot_), giving rise to the characteristic Ru_6f_−O_ot_ pair. This can be seen as the “critical oxidation state” in the RuO_2_ (100) structure, in which the increased presence of adsorbed oxygen on the electrocatalyst surface leads to a reduction in active sites, the rate-limiting factor that leads to reduced overall activity but improved stability. This could, in future studies, serve as a means of stabilizing RuO_2_ for OER.

The degree of electrocatalyst dissolution under corrosive OER conditions has remained a core concern for researchers, and several reports have analyzed this dissolution from different perspectives.^25,37,78–80^ However, there is a paucity of data on the dissolution rates of electrocatalysts in the LOM and AEM pathways. To understand the structural stability of the RuO_2_ catalysts under OER conditions, we quantified Ru dissolution using ICP-MS measurements at 1.9 V vs RHE for 12 h (ref Figure S18). The dissolved Ru metal was calibrated to parts per billion (ppb) and ng/L and normalized to the geometric surface area. The dissolution behavior for all RuO_2_ films in both acidic and alkaline electrolytes appears similar. There is a progressive rate of increase in metal dissolution after OER onset. However, this may vary across different metal types,^80^ as most industrial electrocatalysts operate above the OER onset of RuO_2_ in both acidic and alkaline conditions (1.6−2.0 V vs RHE). From the ratio of the dissolution across the various films (ref Figure 6d), the trend of the dissolution for the RuO_2_ films differing in the level of oxygen becomes apparent, with more than an order of magnitude variation in alkaline conditions and less than 1 order of magnitude in acidic conditions. However, contrary to what may have been reported elsewhere, more dissolution is not observed in alkaline than in acidic conditions. Additionally, an average increase of 2 orders of magnitude in Ru metal dissolution is observed under acidic conditions compared to alkaline conditions.

The Faradaic efficiency (FE) of RuO_2_ corrosion varies over different orders of magnitude as a function of pretreatment and annealing,^19,56,57,80^ dopants infused,^81^ pH of electrolyte,^25^ etc. There is a direct correlation between the degree of dissolution of OER catalysts and their intrinsic activity, even as the rate of dissolution increases progressively with OER onset.^80^ In Figure 6e, the Faradaic selectivity for ruthenium dissolution in the electrolyte, as a percentage of the total electric charge participating in the OER, is shown. The method used to arrive at these numbers is also presented in Supporting Note S1 ^37^ and Figures S19−S20 and compared with some literature values. A tendency toward greater portions of the current density in the less oxygenated films being more due to the dissolution of Ru than in the more oxygenated films is observed, but less than what is recorded for Ru metal (Figure 6e); and this is more pronounced in the acidic conditions than in the alkaline conditions. For the various samples, the Ru dissolution rate, vis-à-vis the Faradaic selectivity toward dissolution, is directly proportional to the OER activity measured by LSV. From the calculations, the Faradaic efficiency for Ru metal dissolution is less than 1% for all RuO_2_ (100) samples, irrespective of deposition parameters and OER mechanism, indicating high electron-utilization efficiency; this compares well with other single-crystal RuO_2_ studies. In the more oxygenated films, Ru dissolution is contained in both conditions, indicating an essential factor in ensuring long-term electrocatalyst stability. Although the general trend of the Faradaic efficiency (FE) is considerably low in the more oxygenated films in both acidic and alkaline conditions, its dissolution rate is about an order of magnitude higher than that of IrO_2_ catalysts,^25,82,83^ making stability concerns the major issue for RuO_2_ OER catalysts in long-term applications. Relating the Faradaic efficiency to the intrinsic site activity vis-à-vis the amount of oxygen molecules evolved per second for the various films, we similarly observe a similar trend of about an order-of-magnitude variation from alkaline to acidic conditions such that the turnover frequency in alkaline conditions is an average of 0.85 s^−1^ at ∼320 mV overpotential which compares very well with the findings of Stoerzinger et al., 2014,^57,84^ similarly the turnover for acidic conditions peaked at ∼9 s^−1^ for less oxygenated samples (ref Supporting Note S2 and Figure S21). Combining the results of high current density, low overpotential, and high FE observed in less oxygenated RuO_2_ films with respect to more oxygenated RuO_2_ films, we hypothesize that a transition from the AEM to the LOM takes place for RuO_2_ samples deposited at 0.0029 and 5 mTorr O_2_, with the remaining samples falling under AEM. However, it should be noted that to propose a definitive OER mechanism via AEM or LOM, direct evidence of lattice oxygen participation is required using operando or product-analysis techniques, including isotope-labeling experiments, differential electrochemical mass spectrometry, DFT calculation and online mass spectrometry.^39,48^

The values presented here were approximately adapted from the works of Roy et al., 2018,^37^ where they compared the various facets of RuO_2_ single crystals PLD-synthesized thin films (001, 101, 111, 110) with powder polycrystalline (PC) RuO_2_, with a Ru dissolution analysis performed using ICP-MS and at CA 1.6 V_RHE_ in 0.05 M H_2_SO_4_ for 2 h. Som et al., 2025 ^71^ on Ru dissolution from molecular beam epitaxy-synthesized RuO_2_ (110) using ICP-MS at CA 1.8 V_RHE_ in 0.1 M HClO4 for 2 h; calculations are presented with ±10% accuracy. Frydendal et al., 2014 ^55^ on Ru dissolution from thermally sputtered thin films using ICP-MS and EQCM at CA 1.8 V_RHE_ in 0.05 M H_2_SO_4_ for 2 h. Tamura et al., 1982 ^85^ on Ru dissolution for thermally prepared hydrous and anhydrous PC RuO_2_ at ∼CP 50 mA cm^−2^ in 0.5 M H_2_SO_4_ using spectrophotometric methods and Ru metal at ∼CP 200 mA cm^−2^. Paoli et al., 2016 ^56^ for Ru dissolution from plasma-treated and annealed RuO_2_ synthesized nanoparticles in 0.05 M H_2_SO_4_ for CV at ∼1.48 V_RHE_. Paoli et al., 2015 ^57^ on Ru dissolution for Ru nanoparticles in 0.05 M H_2_SO_4_ at CV 1.48 V_RHE_ at 10 mV s^−1^. Chang et al. 2014 ^81^ on Ru dissolution for PC Ru metal and faceted SrRuO_3_ films at CV ∼1.3−1.485 V_RHE_ in 0.1 M KOH. Kim et al., 2017 ^86^ on Ru dissolution on PC liquid-feed flame spray synthesized (FSS) SrRuO_3_ at CA 1.2 to 1.6 V_RHE_ in 0.1 M KOH and 0.1 M HClO_4_ in 24 h. Duan et al., 2025 ^87^ on Ru dissolution for hybrid PC Ru/RuS_2_/RuO_2_ synthesized through an in situ combustion reaction followed by calcination at CP 10 mA cm^−2^ in 0.5 m H_2_SO_4_ in >600 h. Du et al., 2025 ^88^ on Ru dissolution for Ru_8_IrO_*x*_ prepared via the sol−gel method at room temperature at CP 100 mA cm^−2^ in 0.5 m H_2_SO_4_ in >800 h. Zhu et al., 2023 ^89^ on Ru dissolution for Ru_0.5_Ir_0.5_O_2_ layered structure prepared via a two-step molten-alkali process at CP 100 mA cm^−2^ in 0.5 M H_2_SO_4_ in >600 h. Wu et al., 2024 ^90^ on Ru dissolution for RuO_2_/CeO_2_@C synthesized by hydrothermal and thermal treatment at CP 10 mA cm^−2^ in 0.5 M H_2_SO_4_ in 100 h. Zhuo et al. 2023 ^91^ on Ru dissolution for Co_3_O_4_/RuO_2_−C prepared by hydrothermal synthesis followed by thermal conversion and acid leaching at CP 10 mA cm^−2^ in 0.1 M HClO_4_. Hu et al.^92^ on metal dissolution in M-RuIrFeCoNiO_2_ prepared by molten-salt-assisted synthesis (MSS) at CP 10 mA cm^−2^ in 0.5 M H_2_SO_4_.

## 4 Conclusions

In conclusion, this study presents a detailed analysis of the thin-film PLD synthesis of epitaxial RuO_2_ (100) OER active electrocatalyst in varying oxygen ambiences looking at the activity-stability compromise. These films were deposited at 0.0029, 5, 25, 50, 75, and 100 mTorr O_2_ conditions, with all samples maintaining epitaxial growth. From the XPS analysis, we deconvolute spectra related to the Ru^4+^-RuO_2_ (lower binding energy, LBE RuO_2_) and the Ru^4+x^-RuO_2+x_ (higher binding energy, HBE RuO_2_), which could be responsible for the formation of soluble, volatile, and high-valence (>4) species such as RuO_3_, perruthenates, or ruthenium tetroxide (RuO_4_). Electrochemical evaluation of the RuO_2_ films for the OER shows higher activity and lower overpotential for less oxygenated films than more oxygenated films; with overpotential at 1 mA cm^−2^ recorded to be between 370 and 476 mV in alkaline conditions and 308−399 mV in acidic conditions. Stability analysis also reveals that, in alkaline conditions, the less oxygenated samples experience a ∼16.5% reduction in current density, while the more oxygenated ones experience a ∼9% reduction after 3 h. In acidic conditions, the less oxygenated samples showed a higher current reduction of ∼21%. Still, the more oxygenated samples showed an improved current output of up to 17%, possibly due to oxychloride formation at elevated potentials. Tafel plots show that the more stoichiometric the sample, the lower the corrosion current and the higher the corrosion potential, confirming that overoxidation of RuO_2_ above stoichiometry does not improve corrosion resistance, as previously reported. The dissolution trend for less oxygenated films and more oxygenated films show more than an order of magnitude variation in alkaline conditions and less than 1 order of magnitude in acidic conditions. But, contrary to what may have been reported elsewhere, more dissolution is not observed in alkaline than in acidic conditions. Additionally, Ru metal dissolution increases by an average of 2 orders of magnitude under acidic conditions compared to alkaline conditions. Faradaic efficiency (FE) was considerably low for the more oxygenated samples under both acidic and alkaline conditions; however, their dissolution rate is about an order of magnitude higher than that of IrO_2_ catalysts reported in the literature.

## Supplementary Material



## Notes

Additional figures, tables, and notes; PLD parameters of the 6 RuO_2_thin films; XRD analysis; Au 4f XPS analysis used for binding energy calibration; XPS survey; molecular fractions of the lower- and higher-binding energy RuO_2_, Species molar ratio comparison across the Ru 3p, Ru 3d, and O 1s spectra; high-resolution XPS and their fitting parameters at the Ru 3d, Ru 3p, and O 1s core levels; cyclic voltammetry curves recorded in 0.1 M KOH and 0.1 M HClO_4_ at various scan rates from 20 to 200 mVs^−1^; overpotential plots in 0.1 M HClO_4_ and 0.1 M KOH; Nyquist plots of the various RuO_2_thin films; corrosion analysis highlighting corrosion current and potential, the anodic and cathodic Tafel slope; electrical double-layer capacitance (EDLC) as a function of scan rate; post-chronoamperometry XPS surface analysis; chronoamperometric analysis of the various RuO_2_films performed at 1.9 V over 12 h.; ICP-MS supporting note; turnover frequency (TOF) supporting note; four-probe resistivity as a function of temperature; AFM images (PDF)

## References

[1] Park J., Lee J. W., Ye B. U., Chun S. H., Joo S. H., Park H., Lee H., Jeong H. Y., Kim M. H., Baik J. M. (2015). Structural evolution of chemically-driven RuO2 nanowires
and 3-dimensional design for photo-catalytic applications. Sci. Rep..

[2] Bianco A., Gradone A., Morandi V., Bergamini G. (2023). RuO2 Nanostructure
as an Efficient and Versatile Catalyst for H2 Photosynthesis. ACS Appl. Energy Mater..

[3] Dresp S., Luo F., Schmack R., Kühl S., Gliech M., Strasser P. (2016). An efficient
bifunctional two-component catalyst for oxygen reduction and oxygen
evolution in reversible fuel cells, electrolyzers and rechargeable
air electrodes. Energy Environ. Sci..

[4] Chen G., Lu R., Zhuang Z., Fei H., Li X., Zhang X., Ma C., Weng J., Wang J., Shang J. (2025). Heterophase
RuO2 oxygen evolution catalyst for durable proton exchange membrane
water electrolysis. Sci. Adv..

[5] Liu X., Yuan Y., Liu J., Liu B., Chen X., Ding J., Han X., Deng Y., Zhong C., Hu W. (2019). Utilizing solar energy to improve the oxygen evolution reaction kinetics
in zinc−air battery. Nat. Commun..

[6] Andersen S. Z., Čolić V., Yang S., Schwalbe J. A., Nielander A. C., McEnaney J. M., Enemark-Rasmussen K., Baker J. G., Singh A. R., Rohr B. A. (2019). A rigorous electrochemical ammonia synthesis protocol
with quantitative isotope measurements. Nature.

[7] Yu Y. Z., Cheng Y., Cheng S., Wu Z. Y. (2025). Advanced Ruthenium-Based
Electrocatalysts for NOx Reduction to Ammonia. Adv. Mater..

[8] Yao Y., Wang H., Yuan X.-Z., Li H., Shao M. (2019). Electrochemical
nitrogen reduction reaction on ruthenium. ACS
Energy Lett..

[9] Wang Y., Li H., Zhou W., Zhang X., Zhang B., Yu Y. (2022). Structurally
disordered RuO2 nanosheets with rich oxygen vacancies for enhanced
nitrate electroreduction to ammonia. Angew.
Chem..

[10] Liu C., Colón B. C., Ziesack M., Silver P. A., Nocera D. G. (2016). Water splitting−biosynthetic
system with CO2 reduction efficiencies exceeding photosynthesis. Science.

[11] Garg S., Li M., Weber A. Z., Ge L., Li L., Rudolph V., Wang G., Rufford T. E. (2020). Advances
and challenges in electrochemical
CO 2 reduction processes: an engineering and design perspective looking
beyond new catalyst materials. J. Mater. Chem.
A.

[12] Karamad M., Hansen H. A., Rossmeisl J., Nørskov J. K. (2015). Mechanistic
pathway in the electrochemical reduction of CO2 on RuO2. ACS Catal..

[13] Deka N., Jones T. E., Falling L. J., Sandoval-Diaz L.-E., Lunkenbein T., Velasco-Velez J.-J., Chan T.-S., Chuang C.-H., Knop-Gericke A., Mom R. V. (2023). On the operando structure of ruthenium
oxides during the oxygen evolution reaction in acidic media. ACS Catal..

[14] Abdullahi Y. Z. (2021). Electronic
and magnetic properties of RuO2 monolayer: DFT+ U investigation. Comput. Condens. Matter.

[15] Exner K. S. (2021). Boosting
the Stability of RuO2 in the Acidic Oxygen Evolution Reaction by Tuning
Oxygen-Vacancy Formation Energies: A Viable Approach Beyond Noble-Metal
Catalysts?. ChemElectrochem.

[16] Weber T., Ortmann T., Escalera-López D., Abb M. J., Mogwitz B., Cherevko S., Rohnke M., Over H. (2020). Visualizing
Potential-Induced Pitting Corrosion of Ultrathin Single-Crystalline
IrO2 (110) Films on RuO2 (110)/Ru (0001) under Electrochemical Water
Splitting Conditions. ChemCatChem.

[17] Over H. (2021). Fundamental
studies of planar single-crystalline oxide model electrodes (RuO2,
IrO2) for acidic water splitting. ACS Catal..

[18] Kasian O., Grote J. P., Geiger S., Cherevko S., Mayrhofer K. J. (2018). The common
intermediates of oxygen evolution and dissolution reactions during
water electrolysis on iridium. Angew. Chem.,
Int. Ed..

[19] Kasian O., Geiger S., Stock P., Polymeros G., Breitbach B., Savan A., Ludwig A., Cherevko S., Mayrhofer K. J. (2016). On the origin of the improved ruthenium stability in
RuO2−IrO2 mixed oxides. J. Electrochem.
Soc..

[20] Lee Y., Suntivich J., May K. J., Perry E. E., Shao-Horn Y. (2012). Synthesis
and activities of rutile IrO2 and RuO2 nanoparticles for oxygen evolution
in acid and alkaline solutions. J. Phys. Chem.
Lett..

[21] Reese A. J., Gelin S., Maalouf M., Wadehra N., Zhang L., Hautier G., Schlom D. G., Dabo I., Suntivich J. (2024). Tracking water
dissociation on RuO2 (110) using atomic force microscopy and first-principles
simulations. J. Am. Chem. Soc..

[22] Kc B. R., Kumar D., Bastakoti B. P. (2024). Block copolymer-mediated synthesis
of TiO2/RuO2 nanocomposite for efficient oxygen evolution reaction. J. Mater. Sci..

[23] Spöri C., Kwan J. T. H., Bonakdarpour A., Wilkinson D. P., Strasser P. (2017). The stability challenges of oxygen evolving catalysts:
towards a common fundamental understanding and mitigation of catalyst
degradation. Angew. Chem., Int. Ed..

[24] Grimaud A., Diaz-Morales O., Han B., Hong W. T., Lee Y.-L., Giordano L., Stoerzinger K. A., Koper M. T., Shao-Horn Y. (2017). Activating
lattice oxygen redox reactions in metal oxides to catalyse oxygen
evolution. Nat. Chem..

[25] Cherevko S., Geiger S., Kasian O., Kulyk N., Grote J.-P., Savan A., Shrestha B. R., Merzlikin S., Breitbach B., Ludwig A. (2016). Oxygen and hydrogen
evolution reactions
on Ru, RuO2, Ir, and IrO2 thin film electrodes in acidic and alkaline
electrolytes: A comparative study on activity and stability. Catal. Today.

[26] Liu Y., Duan T., Xu L., Gao X., Xue L., Xin Y., Ma L., Huang G., Liu T. (2023). Electrocatalyst of
RuO2 decorating TiO2 nanowire arrays for acidic oxygen evolution. Int. J. Hydrogen Energy.

[27] Du K., Zhang L., Shan J., Guo J., Mao J., Yang C.-C., Wang C.-H., Hu Z., Ling T. (2022). Interface
engineering breaks both stability and activity limits of RuO2 for
sustainable water oxidation. Nat. Commun..

[28] Zhang J., Wang X., Zhao X., Chen H., Jia P. (2025). Significantly
Enhanced Acidic Oxygen Evolution Reaction Performance of RuO2 Nanoparticles
by Introducing Oxygen Vacancy with Polytetrafluoroethylene. Polymers.

[29] Zheng Y., Zhou T., Zhang Q., Guo H., Yang J., Ding B., Shao H., Yang X. (2025). Recent advances in
heteroatom-doped RuO2 electrocatalysts for efficient acidic oxygen
evolution reaction. Sci. Technol. Adv. Mater..

[30] Yang J., Ji Y., Shao Q., Zhang N., Li Y., Huang X. (2018). A universal
strategy to metal wavy nanowires for efficient electrochemical water
splitting at pH-universal conditions. Adv. Funct.
Mater..

[31] Zhang L., Jang H., Liu H., Kim M. G., Yang D., Liu S., Liu X., Cho J. (2021). Sodium-decorated amorphous/crystalline
RuO2 with rich oxygen vacancies: a robust pH-universal oxygen evolution
electrocatalyst. Angew. Chem..

[32] Liang J., Zhao Y., Yang C., Zhu S., Wang L., Xu K., Mu X., Han Y., Liu Z., Zhao Z. (2025). Dual-site
cobalt-doped RuO2/TiO2 electrocatalyst enables stable and cost-efficient
acidic oxygen evolution for PEM water electrolysis. J. Am. Chem. Soc..

[33] Lu Q., Liu J., Zou X., Huang B., Wu W., Yin J., Liu Z. Q., Wang Y. (2025). Breaking the Activity-Stability Trade-Off
of RuO2 via Metallic Ru Bilateral Regulation for Acidic Oxygen Evolution
Reaction. Angew. Chem., Int. Ed..

[34] Jiang R., Tran D. T., Li J., Chu D. (2019). Ru@ RuO2 core-shell
nanorods: a highly active and stable bifunctional catalyst for oxygen
evolution and hydrogen evolution reactions. Energy Environ. Mater..

[35] Fu Q., Wong L. W., Zheng X., Tsang C. S., Chen H., Shen W., Ling T., Ly T. H., Deng Q., Zhao J. (2026). The Role of Oxygen
Vacancies: Triggering Lattice Oxygen Oxidation
Mechanism in Acidic OER. Nano Energy.

[36] Stoerzinger K. A., Rao R. R., Wang X. R., Hong W. T., Rouleau C. M., Shao-Horn Y. (2017). The role of
Ru redox in pH-dependent oxygen evolution
on rutile ruthenium dioxide surfaces. Chem.

[37] Roy C., Rao R. R., Stoerzinger K. A., Hwang J., Rossmeisl J., Chorkendorff I., Shao-Horn Y., Stephens I. E. (2018). Trends in activity
and dissolution on RuO2 under oxygen evolution conditions: particles
versus well-defined extended surfaces. ACS Energy
Lett..

[38] Stoerzinger K. A., Diaz-Morales O., Kolb M., Rao R. R., Frydendal R., Qiao L., Wang X. R., Halck N. B., Rossmeisl J., Hansen H. A. (2017). Orientation-dependent
oxygen evolution on RuO2
without lattice exchange. ACS Energy Lett..

[39] Hess F., Rohrlack S., Knapp M., Over H. (2022). Evidence of a tetrahedrally
coordinated RuO4 surface complex on RuO2 (100): Density functional
theory and beyond. J. Phys. Chem. C.

[40] Hess F., Over H. (2023). Coordination inversion of the tetrahedrally coordinated Ru4f surface
complex on RuO2 (100) and its decisive role in the anodic corrosion
process. ACS Catal..

[41] Kim Y. D., Schwegmann S., Seitsonen A. P., Over H. (2001). Epitaxial growth of
RuO2 (100) on Ru (1010): Surface structure and other properties. J. Phys. Chem. B.

[42] Zhang L., Kloppenburg J., Lin C.-Y., Mitrovic L., Gelin S., Dabo I., Schlom D. G., Suntivich J., Hautier G. (2025). Atomistic understanding
of hydrogen coverage on RuO2
(110) surface under electrochemical conditions from ab initio statistical
thermodynamics. J. Phys. Chem. C.

[43] Lin C.-Y., Mitrovic L., Schlom D. G., Suntivich J. (2026). Rate and Mechanisms
of Hydroxyl Dehydrogenation on RuO2 (110) Surfaces. J. Electrochem. Soc..

[44] Jiang S., Ren D., Lei Y., Suo H., Lai W.-H. (2023). Mechanisms of oxygen
evolution reaction in metal oxides: adsorbate evolution mechanism
versus lattice oxygen mechanism. Mater. Lab.

[45] Grimaud A., May K. J., Carlton C. E., Lee Y.-L., Risch M., Hong W. T., Zhou J., Shao-Horn Y. (2013). Double perovskites
as a family of highly active catalysts for oxygen evolution in alkaline
solution. Nat. Commun..

[46] Sun Y., Liao H., Wang J., Chen B., Sun S., Ong S. J. H., Xi S., Diao C., Du Y., Wang J.-O. (2020). Covalency
competition dominates the water oxidation
structure−activity relationship on spinel oxides. Nat. Catal..

[47] Sun J.-X., Liu S.-H., Chen L.-Y., Zhu D.-D., Yu H.-X., Niu Y.-Z., Zhang L.-Q., Li Q.-H., He Y., Miao G.-X. (2025). Accelerating charging and elevating capacity
of TiO2
by interface space charge storage. Rare Met..

[48] Kim I. G., Yim K., Yan L., Shao Y. (2025). Stability of Doped Ruthenium Oxide
as Oxygen Evolution Reaction Electrocatalysts. ACS Appl. Energy Mater..

[49] Gyawali G., Liu M., Chris-Okoro I., Cherono S., Akande W., Barbee B., Nalawade S., Roop J., Pai S., Mantripragada S. (2026). Structural and Electrocatalytic Studies of Pulsed Laser Deposited
Epitaxial RuO2 Thin Films. ACS Appl. Energy
Mater..

[50] Momma K., Izumi F. (2011). VESTA 3 for three-dimensional
visualization of crystal, volumetric
and morphology data. Appl. Crystallogr..

[51] Yeh J., Lindau I. (1985). Atomic subshell
photoionization cross sections and
asymmetry parameters: 1< Z< 103. At. Data
Nucl. Data Tables.

[52] Powell, C. J. ; Jablonski, A. NIST Electron Inelastic-Mean-Free-Path Database 71, Version 1.0; National Institute of Standards and Technology (NIST), 1999.

[53] Tanuma S., Powell C., Penn D. (2005). Calculations
of electron inelastic
mean free paths. Surf. Interface Anal..

[54] Tanuma S., Powell C. J., Penn D. R. (1988). Calculations
of electron inelastic
mean free paths for 31 materials. Surf. Interface
Anal..

[55] Frydendal R., Paoli E. A., Knudsen B. P., Wickman B., Malacrida P., Stephens I. E., Chorkendorff I. (2014). Benchmarking the stability of oxygen
evolution reaction catalysts: the importance of monitoring mass losses. ChemElectrochem.

[56] Paoli E. A., Masini F., Frydendal R., Deiana D., Malacrida P., Hansen T. W., Chorkendorff I., Stephens I. E. (2016). Fine-tuning the
activity of oxygen evolution catalysts: The effect of oxidation pre-treatment
on size-selected Ru nanoparticles. Catal. Today.

[57] Paoli E. A., Masini F., Frydendal R., Deiana D., Schlaup C., Malizia M., Hansen T. W., Horch S., Stephens I. E., Chorkendorff I. (2015). Oxygen evolution
on well-characterized mass-selected
Ru and RuO 2 nanoparticles. Chem. Sci..

[58] Kutsuzawa D., Oka D., Fukumura T. (2020). Thickness Effects on Crystal Growth and Metal−Insulator
Transition in Rutile-Type RuO2 (100) Thin Films. Phys. Status Solidi B.

[59] Liao C.-T., Wang Y.-C., Tien Y.-C., Huang S.-Y., Qu D. (2024). Separation
of inverse altermagnetic spin-splitting effect from inverse spin Hall
effect in RuO 2. Phys. Rev. Lett..

[60] Fatima Z., Oka D., Fukumura T. (2021). Systematic
application of extremely large strain to
rutile-type RuO2 (100) epitaxial thin films on substrates with large
lattice mismatches. Cryst. Growth Des..

[61] Moatti A., Bayati R., Narayan J. (2016). Epitaxial growth of rutile TiO2 thin
films by oxidation of TiN/Si{100} heterostructure. Acta Mater..

[62] Narayan J. (2013). Recent progress
in thin film epitaxy across the misfit scale (2011 Acta Gold Medal
Paper). Acta Mater..

[63] Narayan J., Larson B. C. (2003). Domain epitaxy:
A unified paradigm for thin film growth. J.
Appl. Phys..

[64] Morgan D. J. (2015). Resolving
ruthenium: XPS studies of common ruthenium materials. Surf. Interface Anal..

[65] Zhang M., Chen W., Ding S.-J., Liu Z.-Y., Huang Y., Liao Z.-W., Zhang D. W. (2008). Physical
and electrical characterization
of atomic-layer-deposited Ru nanocrystals embedded into Al2O3 for
memory applications. J. Phys. D: Appl. Phys..

[66] Nalawade S., Kim R. S., Mahl J., Cherono S., Chris-Okoro I., Craciun V., Yano J., Crumlin E., Kumar D., Aravamudhan S. (2026). Phase Transformation
and Water Adsorption Behavior
of As-Deposited and Annealed Ru Metal Thin Films Prepared by Atomic
Layer Deposition. Adv. Mater. Interfaces.

[67] Nalawade S., Vondee E., Liu M., Chris-Okoro I., Cherono S., Kumar D., Aravamudhan S. (2025). Role of Oxygen
Concentration in Reactive Sputtering of RuO2 Thin Films: Tuning Surface
Chemistry for Enhanced Electrocatalytic Performance. Crystals.

[68] Allhusen J. S., Conboy J. C. (2013). Preparation and Characterization of Conductive and
Transparent Ruthenium Dioxide Sol−Gel Films. ACS Appl. Mater. Interfaces.

[69] Angermann M., Jakopic G., Prietl C., Griesser T., Reichmann K., Deluca M. (2023). Highly conductive RuO2
thin films from novel facile
aqueous chemical solution deposition. J. Sol
Gel Sci. Technol..

[70] Rochefort D., Dabo P., Guay D., Sherwood P. (2003). XPS investigations
of thermally prepared RuO2 electrodes in reductive conditions. Electrochim. Acta.

[71] Som J., Reese A. J., Mitrovic L., Kim R. S., McBride S., Nalawade S. S., Aravamudhan S., Hautier G., Yano J., Schlom D. G. (2025). Strained-Induced
Morphological Reconstruction of RuO2
(110) Thin-Film Electrocatalysts. J. Am. Chem.
Soc..

[72] Rossmeisl J., Qu Z.-W., Zhu H., Kroes G.-J., Nørskov J. K. (2007). Electrolysis
of water on oxide surfaces. J. Electroanal.
Chem..

[73] Soni A., Maurya S. K., Malviya M. (2025). Exploring
electrocatalysts for oxygen
evolution: A comprehensive comparative review in alkaline and acidic
medium. J. Power Sources.

[74] Suen N.-T., Hung S.-F., Quan Q., Zhang N., Xu Y.-J., Chen H. M. (2017). Electrocatalysis
for the oxygen evolution reaction:
recent development and future perspectives. Chem. Soc. Rev..

[75] Yoo J. S., Rong X., Liu Y., Kolpak A. M. (2018). Role of lattice
oxygen participation in understanding trends in the oxygen evolution
reaction on perovskites. ACS Catal..

[76] Shi Z., Li J., Wang Y., Liu S., Zhu J., Yang J., Wang X., Ni J., Jiang Z., Zhang L. (2023). Customized reaction
route for ruthenium oxide towards stabilized
water oxidation in high-performance PEM electrolyzers. Nat. Commun..

[77] Zhao Y., Hu J., Chiang C.-L., Li Y., Yang W., Yang Z., Hung W.-H., Lin Y.-G., Chen Z., Li B. (2022). Ruthenium oxychloride
supported by manganese oxide for stable oxygen
evolution in acidic media. J. Mater. Chem. A.

[78] Li Z., Dang N., Sweers M. E., Wang X., Kariuki N., Park J. H., Kropf A. J., Chuang A., Ilavsky J., Xu W. (2026). Composition,
Activity, and Stability of IrO_x_ Oxygen Evolution Reaction
Electrocatalysts. ACS Catal..

[79] Danilovic N., Subbaraman R., Chang K.-C., Chang S. H., Kang Y. J., Snyder J., Paulikas A. P., Strmcnik D., Kim Y.-T., Myers D. (2014). Activity−Stability
Trends for the Oxygen Evolution
Reaction on Monometallic Oxides in Acidic Environments. J. Phys. Chem. Lett..

[80] Cherevko S., Zeradjanin A. R., Topalov A. A., Kulyk N., Katsounaros I., Mayrhofer K. J. (2014). Dissolution of noble metals during oxygen evolution
in acidic media. ChemCatChem.

[81] Chang S. H., Danilovic N., Chang K.-C., Subbaraman R., Paulikas A. P., Fong D. D., Highland M. J., Baldo P. M., Stamenkovic V. R., Freeland J. W. (2014). Functional links between
stability and reactivity of strontium ruthenate single crystals during
oxygen evolution. Nat. Commun..

[82] Geppert J., Röse P., Pauer S., Krewer U. (2022). Microkinetic barriers
of the oxygen evolution on the oxides of iridium, ruthenium and their
binary mixtures. ChemElectroChem.

[83] Naraki N., Okayama Y., Todoroki N. (2025). Surface orientation
dependent electrochemical
stability of RuO2 and IrO2 under acidic oxygen evolution reaction. Electrochem. Commun..

[84] Stoerzinger K. A., Qiao L., Biegalski M. D., Shao-Horn Y. (2014). Orientation-dependent
oxygen evolution activities of rutile IrO2 and RuO2. J. Phys. Chem. Lett..

[85] Tamura H., Iwakura C. (1982). Metal oxide anodes
for oxygen evolution. Int. J. Hydrogen Energy.

[86] Kim B.-J., Abbott D. F., Cheng X., Fabbri E., Nachtegaal M., Bozza F., Castelli I. E., Lebedev D., Schäublin R., Copéret C. (2017). Unraveling thermodynamics, stability, and oxygen
evolution activity of strontium ruthenium perovskite oxide. ACS Catal..

[87] Duan X., Wen N., Liu S., Li H., Jiao X., Chen D., Xia Y. (2025). Lattice strain engineering
in Ru-based electrocatalysts for efficient
acidic overall water splitting and Ru dissolution suppression. ACS Catal..

[88] Du X., Sun Y., Cui J., Wu C., Yang X., Chen X., Tao H., Liu L., Ye J. (2025). Covalency Regulation of Ru-Based
Solid Solutions by Iridium for Durable Proton Exchange Membrane Water
Electrolysis at 2 A cm− 2. Adv. Funct.
Mater..

[89] Zhu W., Song X., Liao F., Huang H., Shao Q., Feng K., Zhou Y., Ma M., Wu J., Yang H. (2023). Stable and oxidative
charged Ru enhance the acidic
oxygen evolution reaction activity in two-dimensional ruthenium-iridium
oxide. Nat. Commun..

[90] Wu Y., Yao R., Zhang K., Zhao Q., Li J., Liu G. (2024). RuO2/CeO2
heterostructure anchored on carbon spheres as a bifunctional electrocatalyst
for efficient water splitting in acidic media. Chem. Eng. J..

[91] Zhou F., Chen J., Yang Y., Ke X., Liu X., Zhang L., Li J., Jin H., Wang S., Li Y. (2023). Exceptionally active and stable RuO2 by constructing
p-n heterojunction between Co3O4 and RuO2 for acidic water oxidation. Appl. Surf. Sci..

[92] Hu C., Yue K., Han J., Liu X., Liu L., Liu Q., Kong Q., Pao C.-W., Hu Z., Suenaga K. (2023). Misoriented high-entropy iridium ruthenium
oxide for acidic water
splitting. Sci. Adv..

