# Supplementary material for: Effect of Varying RuO2 (100) Thin-Film Stoichiometry on the Activity-Stability Compromise in the Oxygen Evolution Reaction: Pathway to RuO2 Degradation
Source: ACS Omega. 2026 Sep 19;11(39):60039–52. doi: 10.1021/acsomega.6c07471 (PMC13648090; doi:10.1021/acsomega.6c07471)
Supplement: Supplementary file 1 [file ao6c07471_si_001.pdf]

Supporting Information for Publication

Effect of Varying RuO_2 (100) Thin Film Stoichiometry on the Activity-Stability Compromise in Oxygen Evolution Reaction– Pathway to RuO_2 Degradation

Ikenna Chris-Okoro[1], Ghanashyam Gyawali[1], Sheilah Cherono[1], Mengxin Liu[1], Wisdom Akande[1], Swapnil Nalawade[2], Brianna Barbee[1], Shobha Mantripragada[2], Shyam Aravamudhan[2], Veluchamy Palaniappagounder[1] and Dhananjay Kumar[1]

[1] Department of Mechanical Engineering, North Carolina A&T State University, Greensboro, NC 27411, USA

[2] Joint School of Nanoscience and Nanoengineering, North Carolina A&T State University, Greensboro, NC 27401, USA

Correspondence: dkumar@ncat.edu

Pulses	Freq. (Hz)	Deposition Temp. (°C)	Deposition O_2 Pressure (mTorr)	Post-Deposition Cooling (Torr)	Laser Energy (J.cm^{-2})	Sheet Resistance (Ω/Square)
5000	10	600	0.0029	>50	2.3	1.61
5000	10	600	5	>50	2.28	4.50
5000	10	600	25	>50	2.3	5.71
5000	10	600	50	>50	2.32	7.19
5000	10	600	75	>50	2.28	8.70
5000	10	600	100	>50	2.26	8.28

Table S1. Pulsed laser deposition (PLD) parameters of the six RuO_2 thin films.

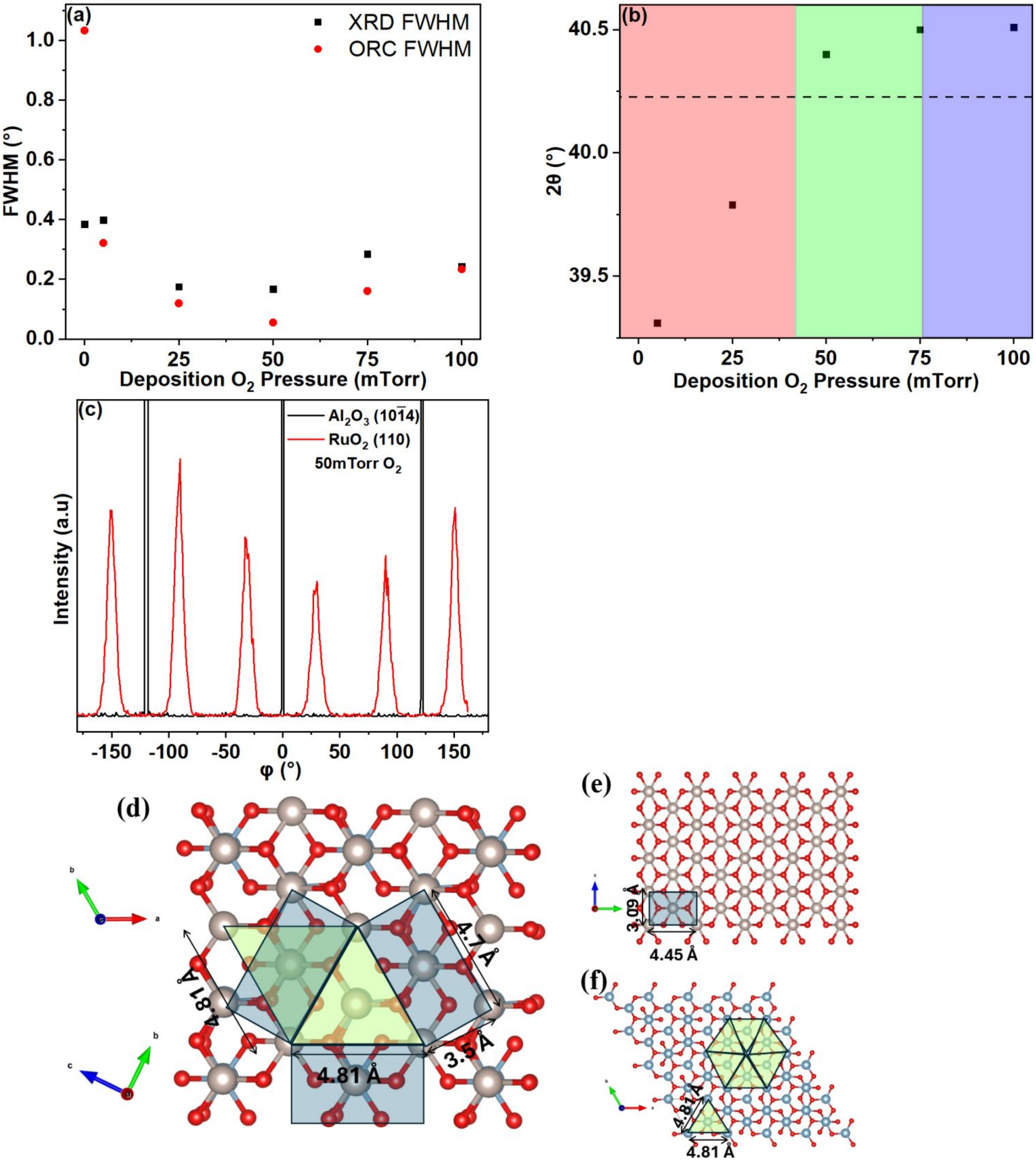

Figure S1. (a) Variation of the FWHM from both the XRD and ORC as a function of the deposition pressure, (b) The correspondence of 2θ position with stoichiometry of RuO_2 (Red-Sub stoichiometric, green- stoichiometric, blue-hyper stoichiometric), (c) Phi-scan measurement, and (d-f) Schematic illustration of the epitaxial relationship between the rutile RuO_2 and hexagonal Al_2O_3 at the substrate-film interface. The red indicates oxygen atoms, the blue indicates aluminum atoms, and the grey indicates ruthenium atoms.

Deposition O_2 Pressure (mTorr)	Thickness (nm)	2theta (°)		XRD FWHM (°)		ORC FWHM (°)	Resistivity (μΩ.cm)	Density (g.cm^{-3})	Lattice Parameter (Å)	Roughness (nm)
0.0029	55.35	42.15	92.10	0.384	0.453	1.032	8.91	9.159		2.708
5	78.29	39.31	84.55	0.399	0.878	0.321	35.25	7.311	4.58	2.369
25	78.05	39.79	85.65	0.175	0.311	0.119	44.57	7.527	4.53	2.047
50	79.49	40.40	87.50	0.167	0.279	0.055	57.12	7.462	4.47	2.088
75	79.26	40.50	87.61	0.285	0.324	0.161	68.99	7.622	4.45	3.239
100	82.87	40.51	87.63	0.243	0.423	0.233	68.36	7.611	4.45	3.050

Table S2. XRD analysis showing the film thickness, 2-theta position, resistivity, density, lattice parameters, and roughness.

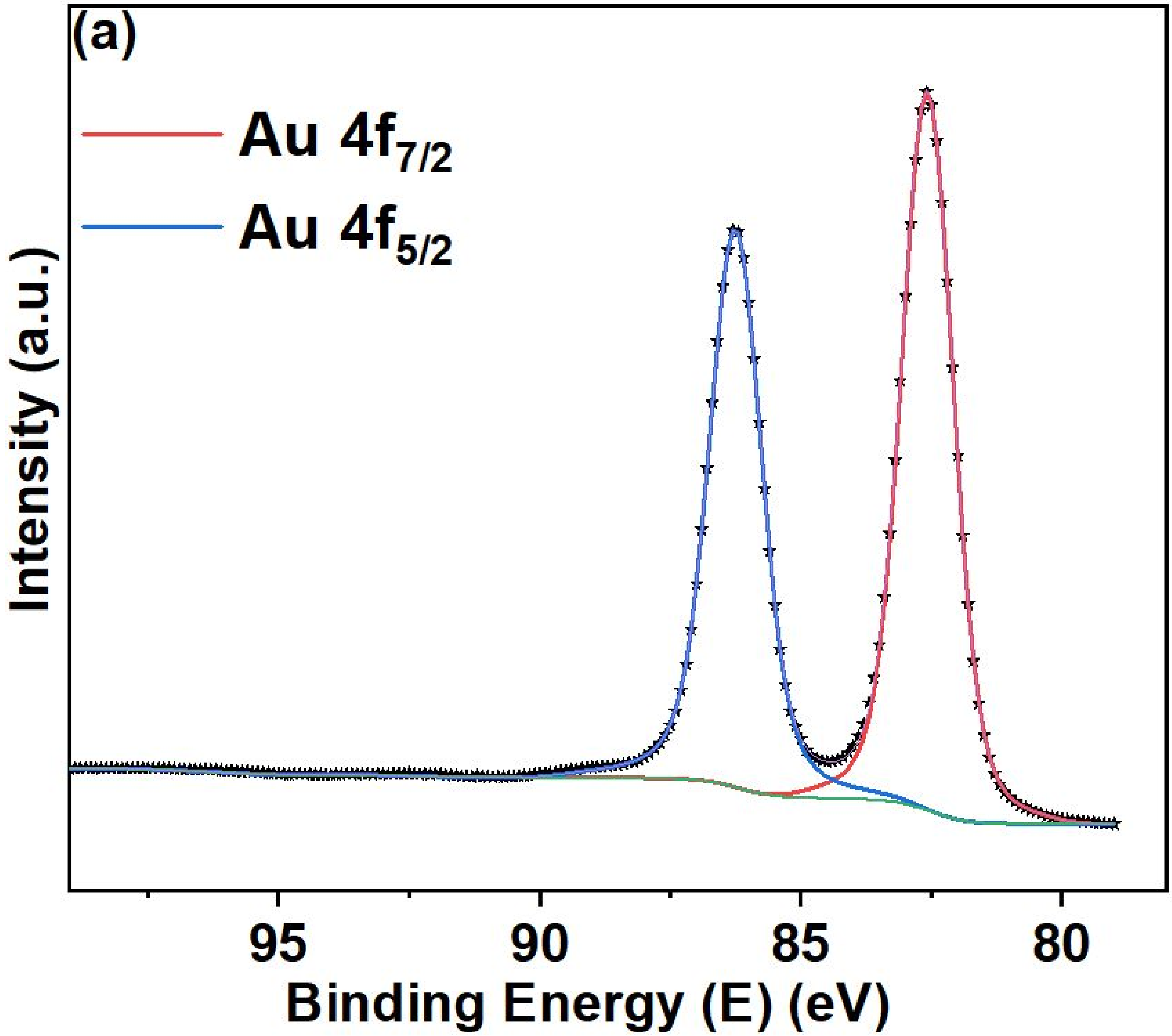

Figure S2. Representation of the High-Resolution Au 4f XPS analysis used for binding energy calibration.

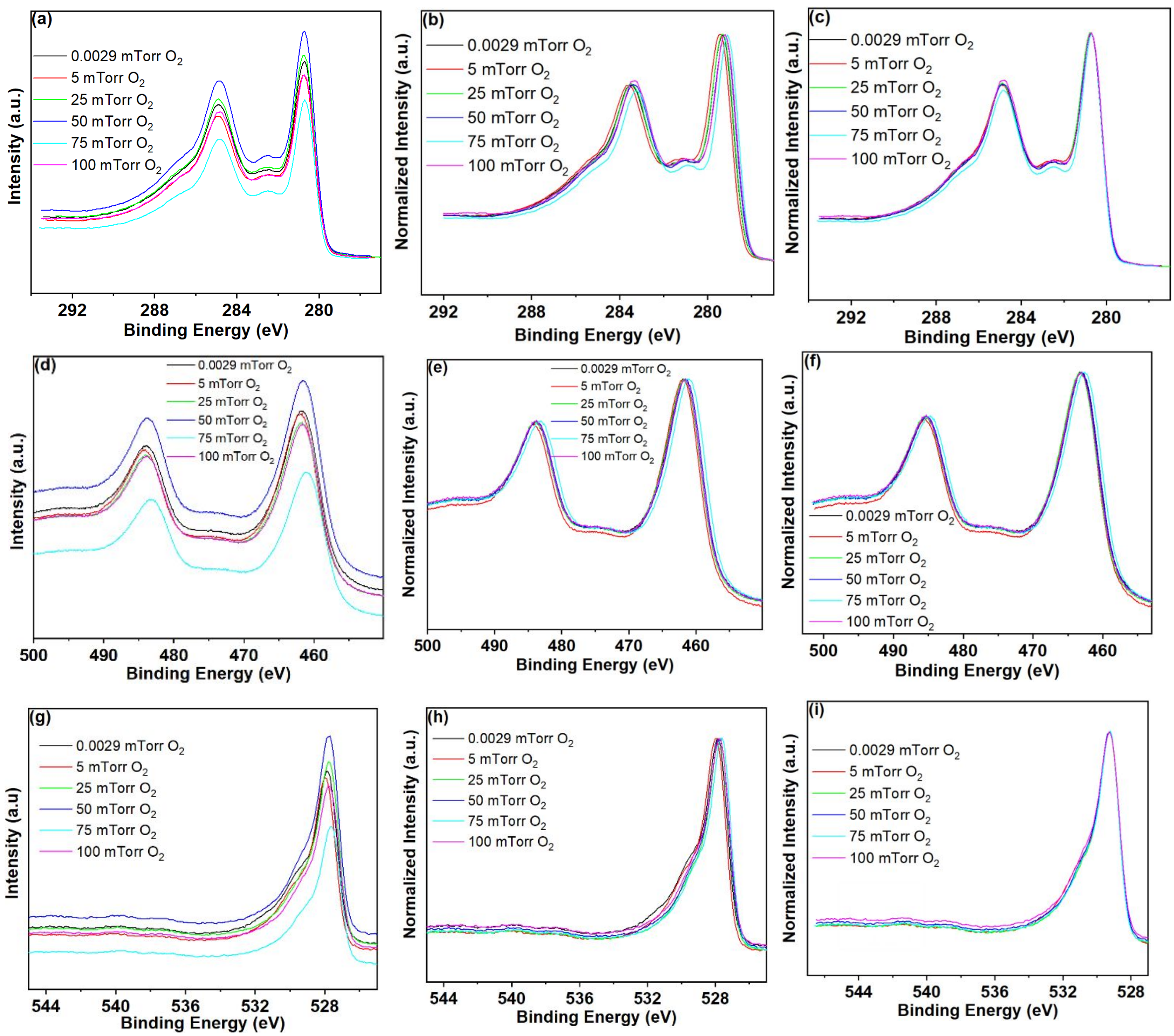

Figure S3. High-Resolution XPS data for Ru 3d, Ru 3p, O 1s; (a, d, g) as initially derived; (b, e, h) normalized; (c, f, i) shifted with respect to the Au 4f spectra.

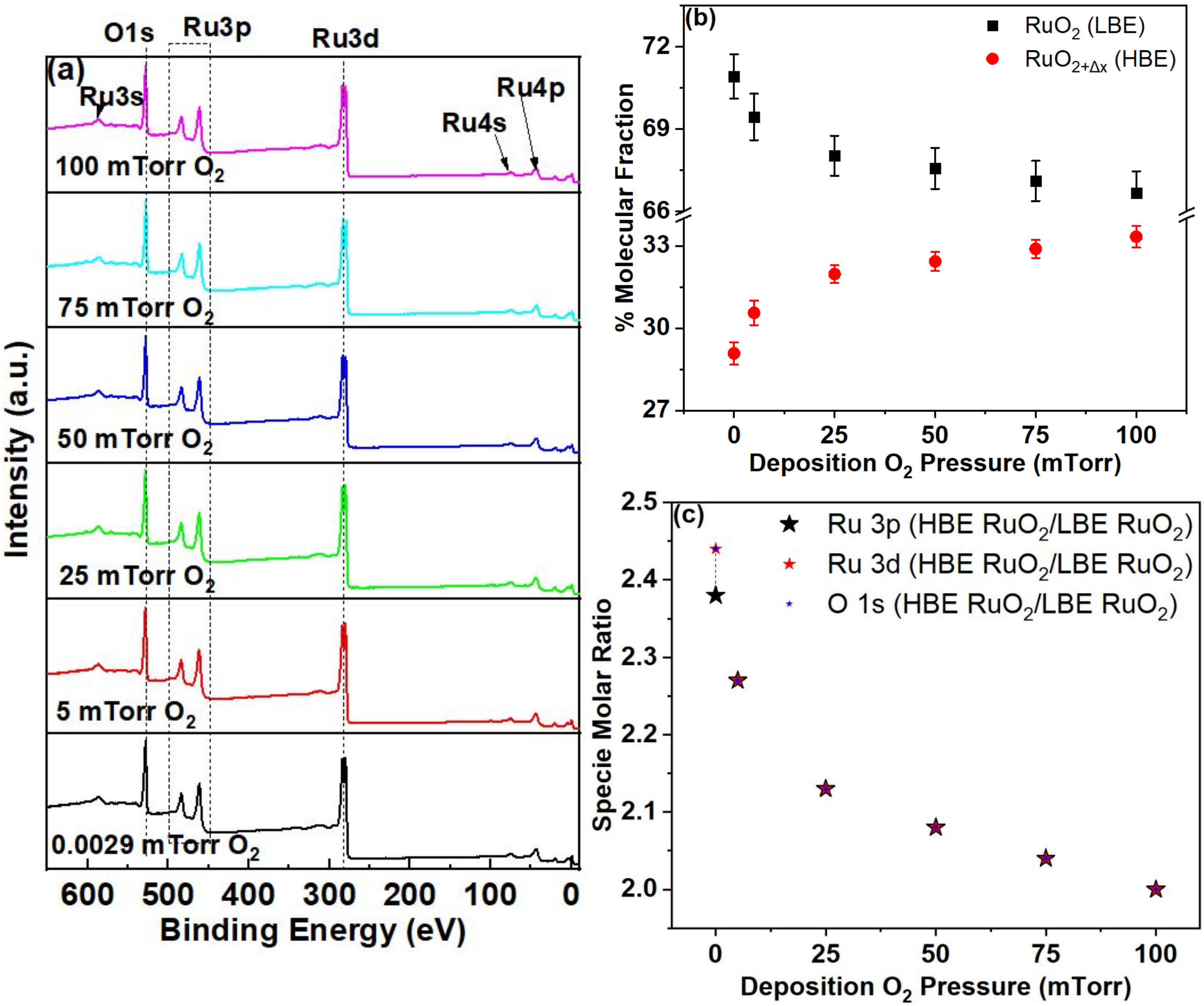

Figure S4. (a) XPS Survey highlighting the core level lines originating from Ru (Ru 4s, Ru 4p, Ru 3s, Ru 3p, and Ru 3d), and O 1s (b) Molecular fraction of the lower and higher binding energy RuO_2 (c) Specie molar ratio comparison across the Ru 3p, Ru 3d, O 1s spectra (the separation of red and black stars indicates accuracy of fit/error bar).

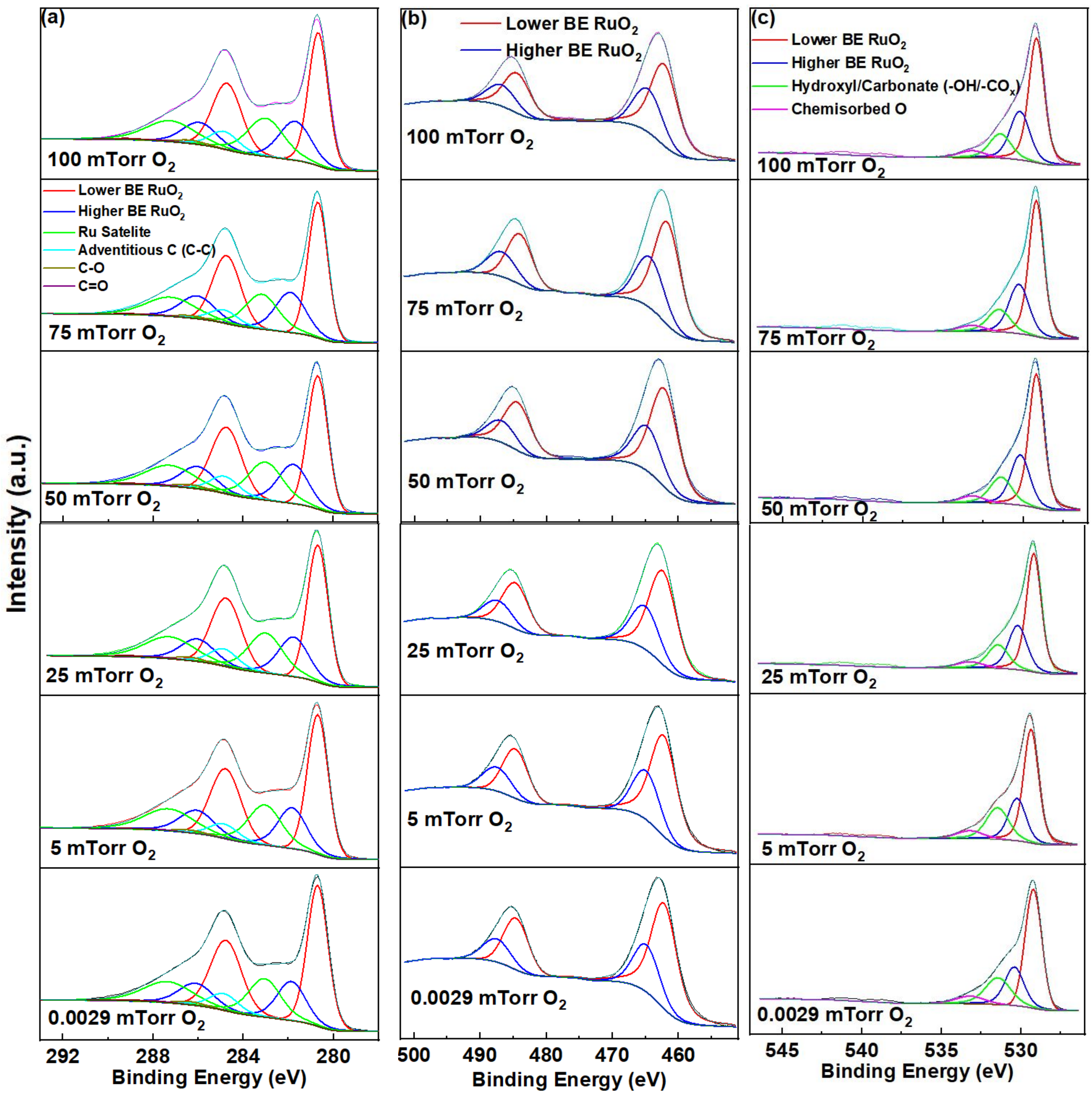

Figure S5. High-resolution XPS (a) Ru 3d core level, (b) Ru 3p core level, and (c) O 1s core level.

**Ru 3d**	Binding Energy/eV	FWHM/eV
LBE RuO_2 5/2	280.615 ± 0.110	1.03 ± 0.06
LBE RuO_2 3/2	284.755 ± 0.135	1.57 ± 0.13
HBE RuO_2 5/2	281.785 ± 0.105	1.62 ± 0.19
HBE RuO_2 3/2	285.813 ± 0.170	1.75 ± 0.05
RuO_2 Satellite 5/2	283.085 ± 0.185	1.82 ± 0.27
RuO_2 Satellite 3/2	287.300 ± 0.260	2.27 ± 0.35
Adventitious C (C-C)	284.835 ± 0.015	1.49 ± 0.01
C-O	286.325 ± 0.025	1.25 ± 0.15
C=O	287.800 ± 0.050	1.39 ± 0.11
O-C=O	288.975 ± 0.075	1.49 ± 0.01

Table S3. Fitting parameters for Binding Energies (BE) and Full Width of Half Maxima (FWHM) in the Ru 3d Spectra.

**Ru 3p**	Binding Energy/eV	FWHM/eV
LBE RuO_2 5/2	462.415 ± 0.275	4.45 ± 0.44
LBE RuO_2 3/2	484.370 ± 0.300	4.57 ± 0.49
HBE RuO_2 5/2	464.705 ± 0.365	4.89 ± 0.25
HBE RuO_2 3/2	487.170 ± 0.360	5.06 ± 0.05

Table S4. Fitting parameters for Binding Energies (BE) and Full Width of Half Maxima (FWHM) in the Ru 3p Spectra.

**O 1s**	Binding Energy/eV	FWHM/eV
LBE RuO_2	529.250 ± 0.170	1.18 ± 0.12
HBE RuO_2	530.350 ± 0.150	1.42 ± 0.19
Hydroxyl/Carbonate (-OH/-CO_x)	531.615± 0.165	1.74 ± 0.18
Chemisorbed O	533.252 ± 0.180	1.71 ± 0.22

Table S5. Fitting parameters for Binding Energies (BE) and Full Width of Half Maxima (FWHM) in the O 1s Spectra.

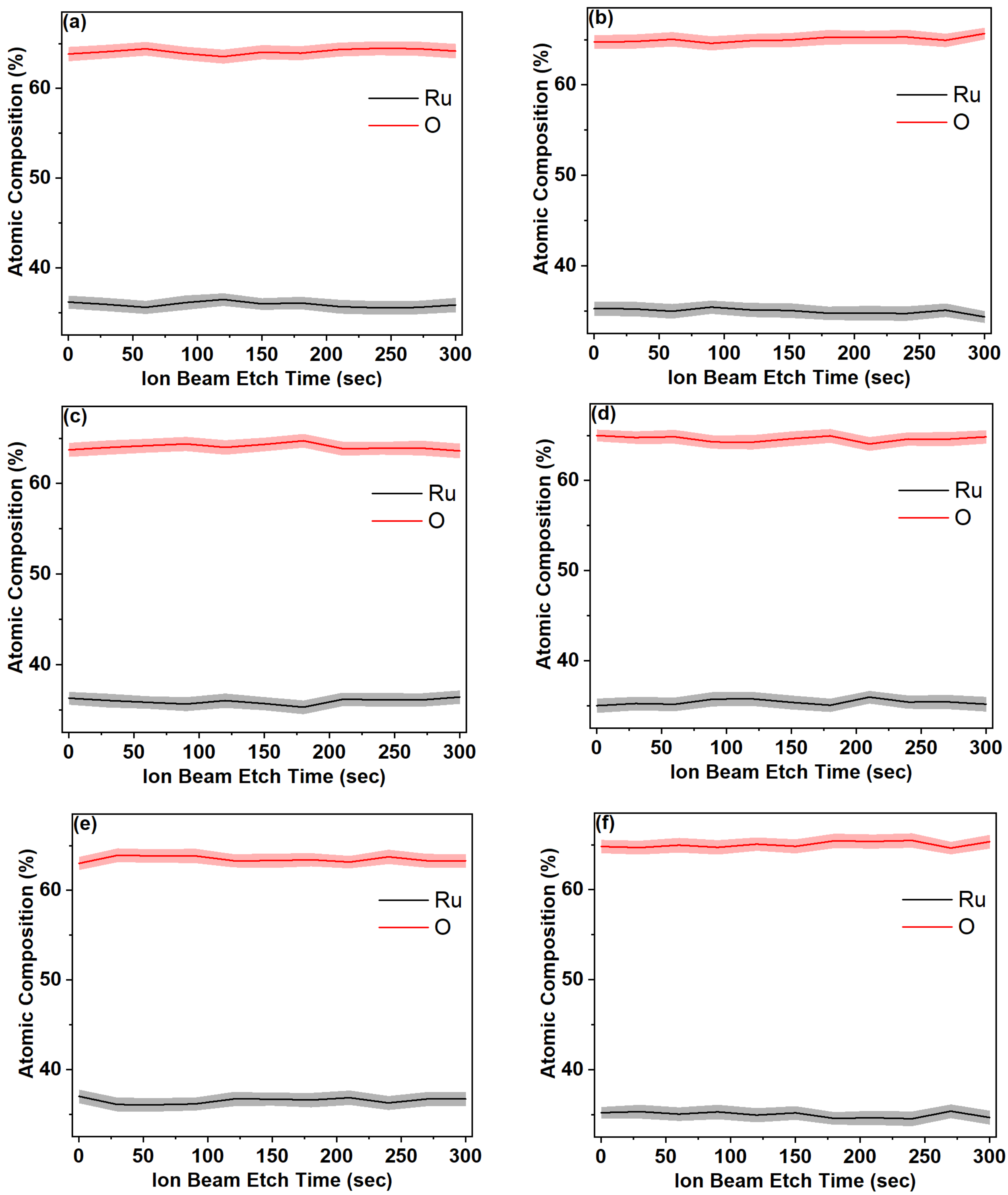

Figure S6. Elemental composition from XPS depth profile recorded at an interval of 30s showing the constancy of Ru and O for (a) 0.0029 mTorr O_2 (b) 5 mTorr O_2 (c) 25 mTorr O_2 (d) 50 mTorr O_2 (e) 75 mTorr O_2 (f) 100 mTorr O_2

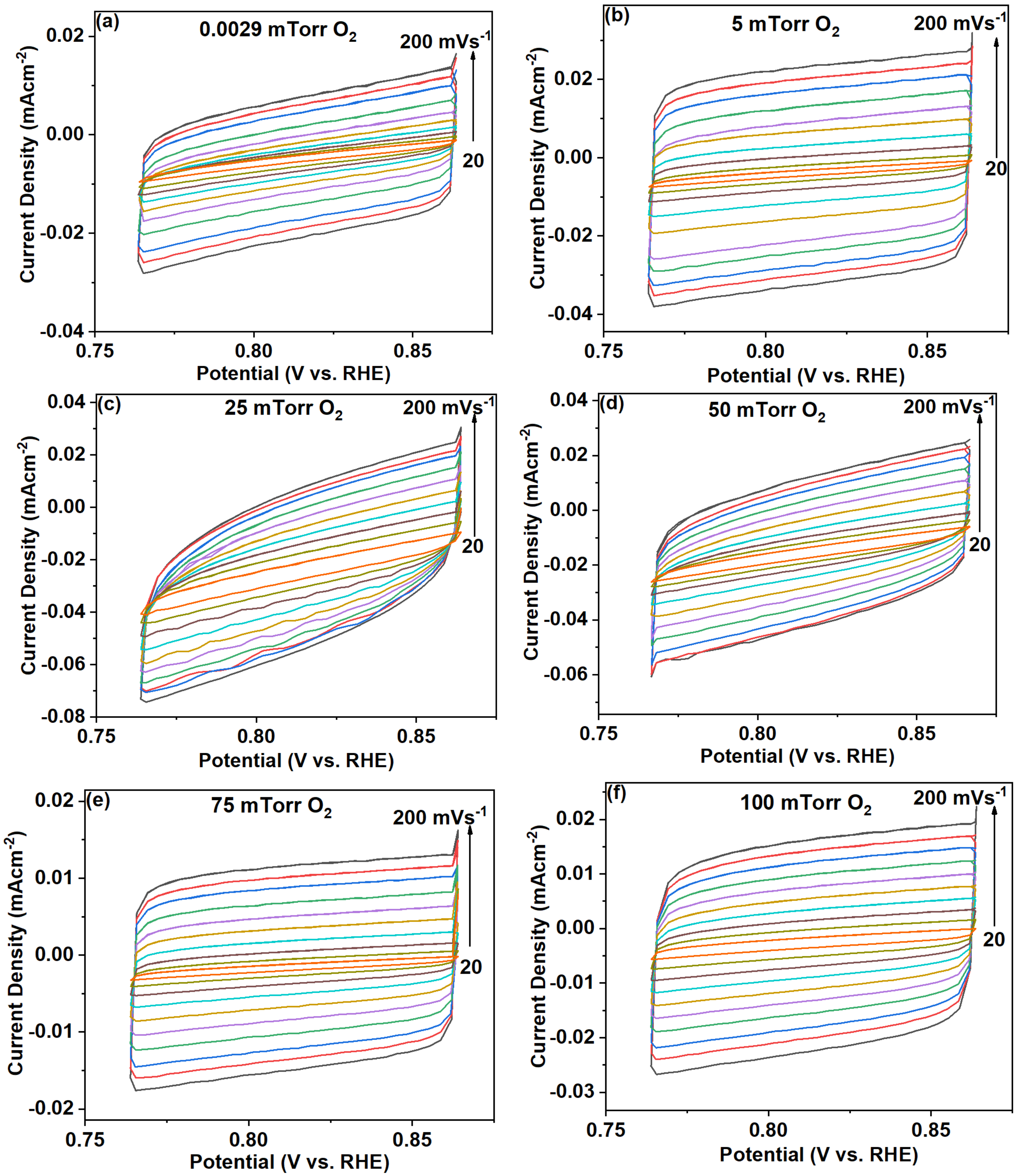

Figure S7. Cyclic voltammetry curves recorded in 0.1 M KOH at various scan rates from 20 to 200 mVs^{-1} in the capacitive region for (a) 0.0029 mTorr O_2 (b) 5 mTorr O_2 (c) 25 mTorr O_2 (d) 50 mTorr O_2 (e) 75 mTorr O_2 (f) 100 mTorr O_2 RuO_2 thin film samples.

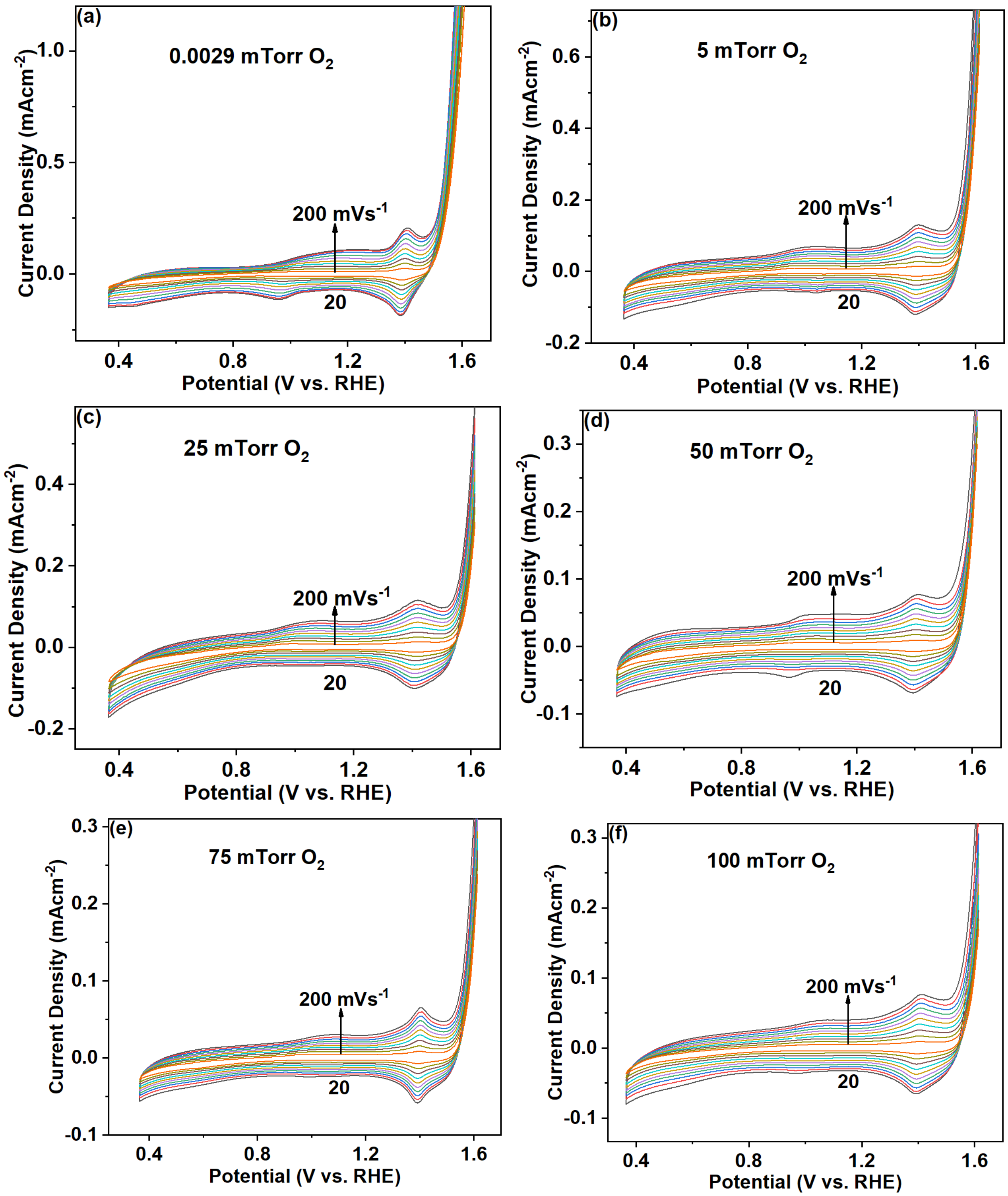

Figure S8. Cyclic voltammetry curves recorded in 0.1 M KOH at various scan rates from 20 to 200 mVs^{-1} for (a) 0.0029 mTorr O_2 (b) 5 mTorr O_2 (c) 25 mTorr O_2 (d) 50 mTorr O_2 (e) 75 mTorr O_2 (f) 100 mTorr O_2 RuO_2 thin film sample.

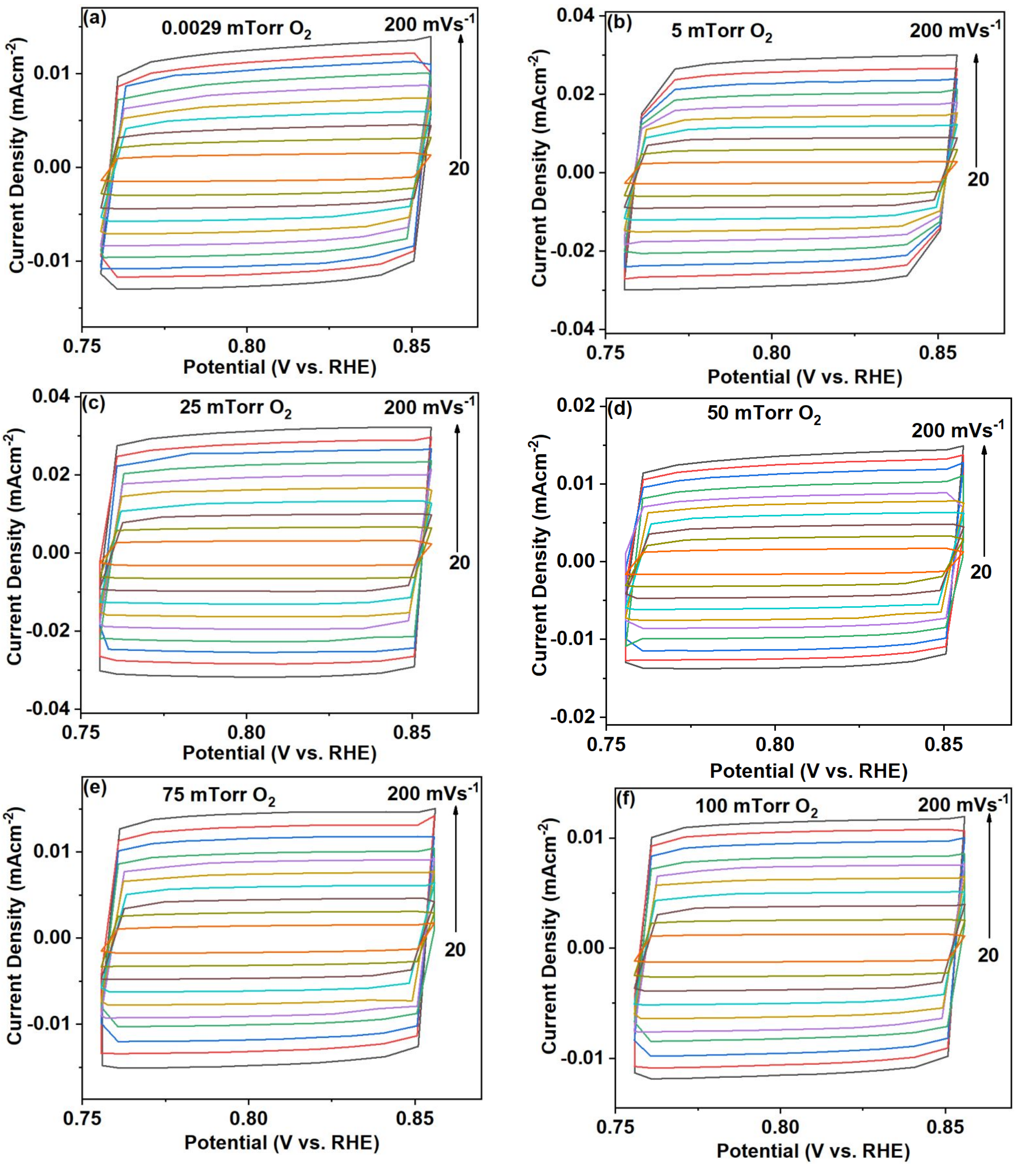

Figure S9. Cyclic voltammetry curves recorded in 0.1 M $HClO_4$ at various scan rates from 20 to 200 mVs^{-1} in the capacitive region for (a) 0.0029 mTorr O_2 (b) 5 mTorr O_2 (c) 25 mTorr O_2 (d) 50 mTorr O_2 (e) 75 mTorr O_2 (f) 100 mTorr O_2 RuO_2 thin film sample.

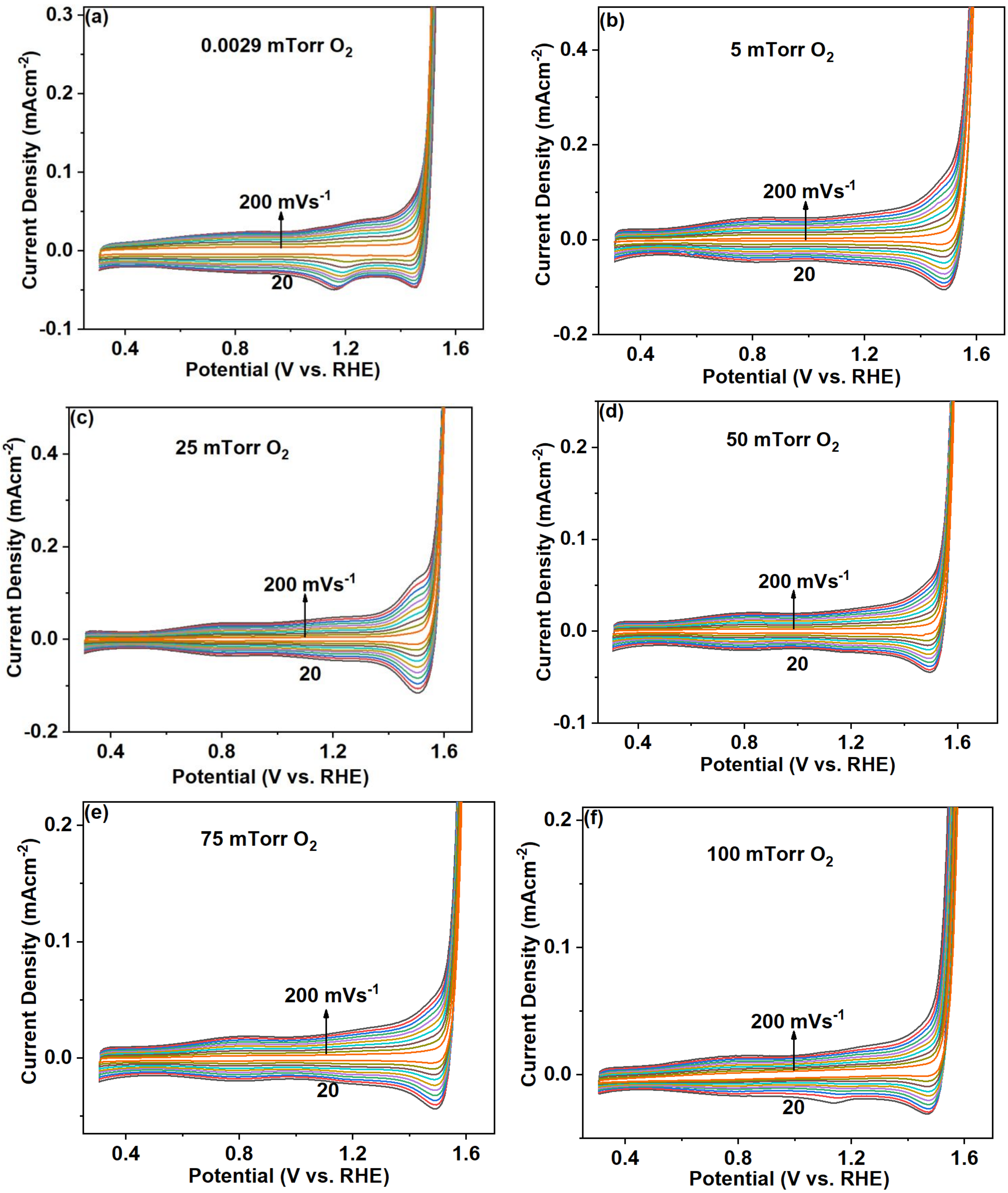

Figure S10. Cyclic voltammetry curves recorded in 0.1 M $HClO_4$ at various scan rates from 20 to 200 mVs^{-1} for (a) 0.0029 mTorr O_2 (b) 5 mTorr O_2 (c) 25 mTorr O_2 (d) 50 mTorr O_2 (e) 75 mTorr O_2 (f) 100 mTorr O_2 RuO_2 thin film sample.

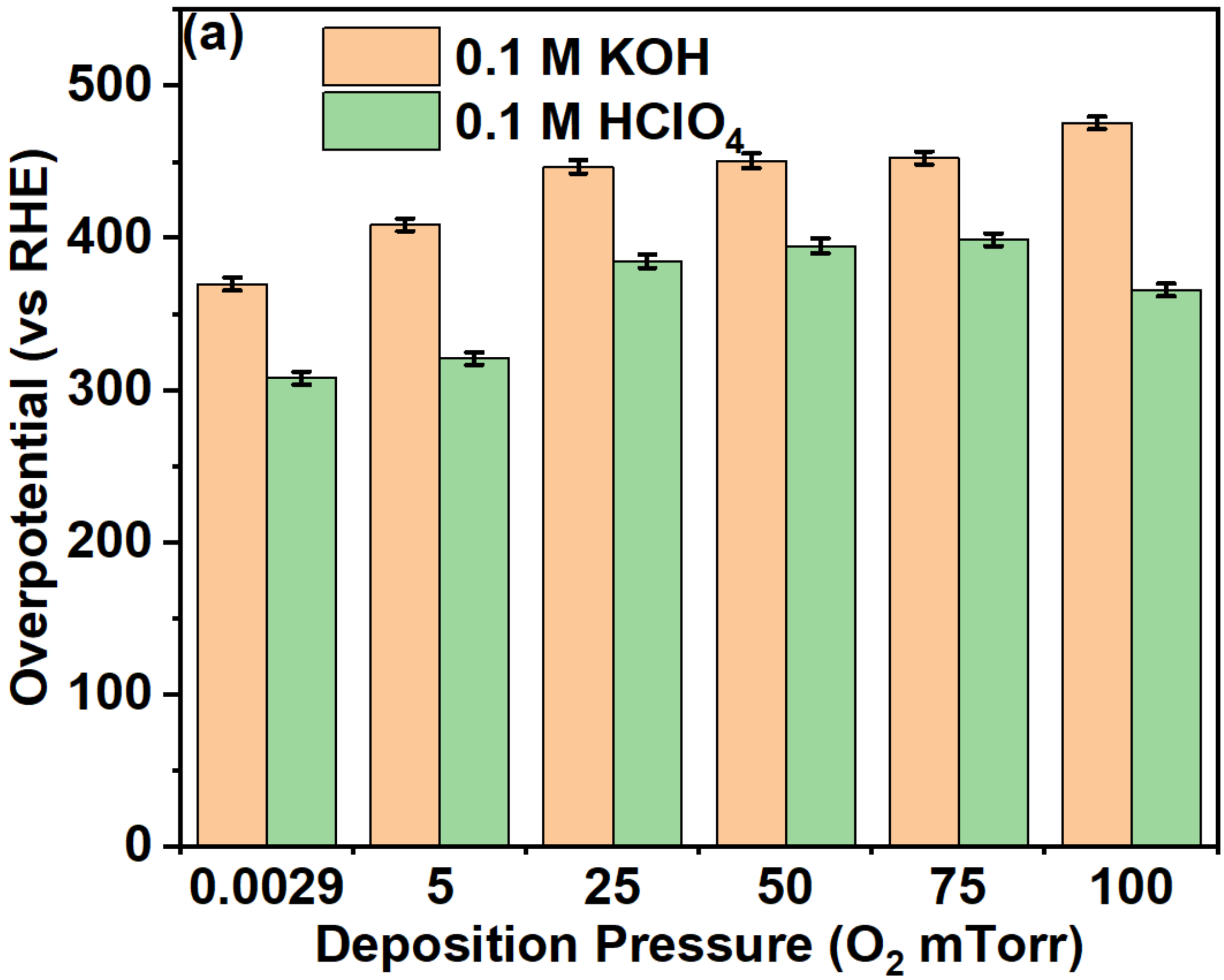

Figure S11. Overpotential plots in 0.1 M $HClO_4$ and 0.1 M KOH as a function of oxygen deposition pressure.

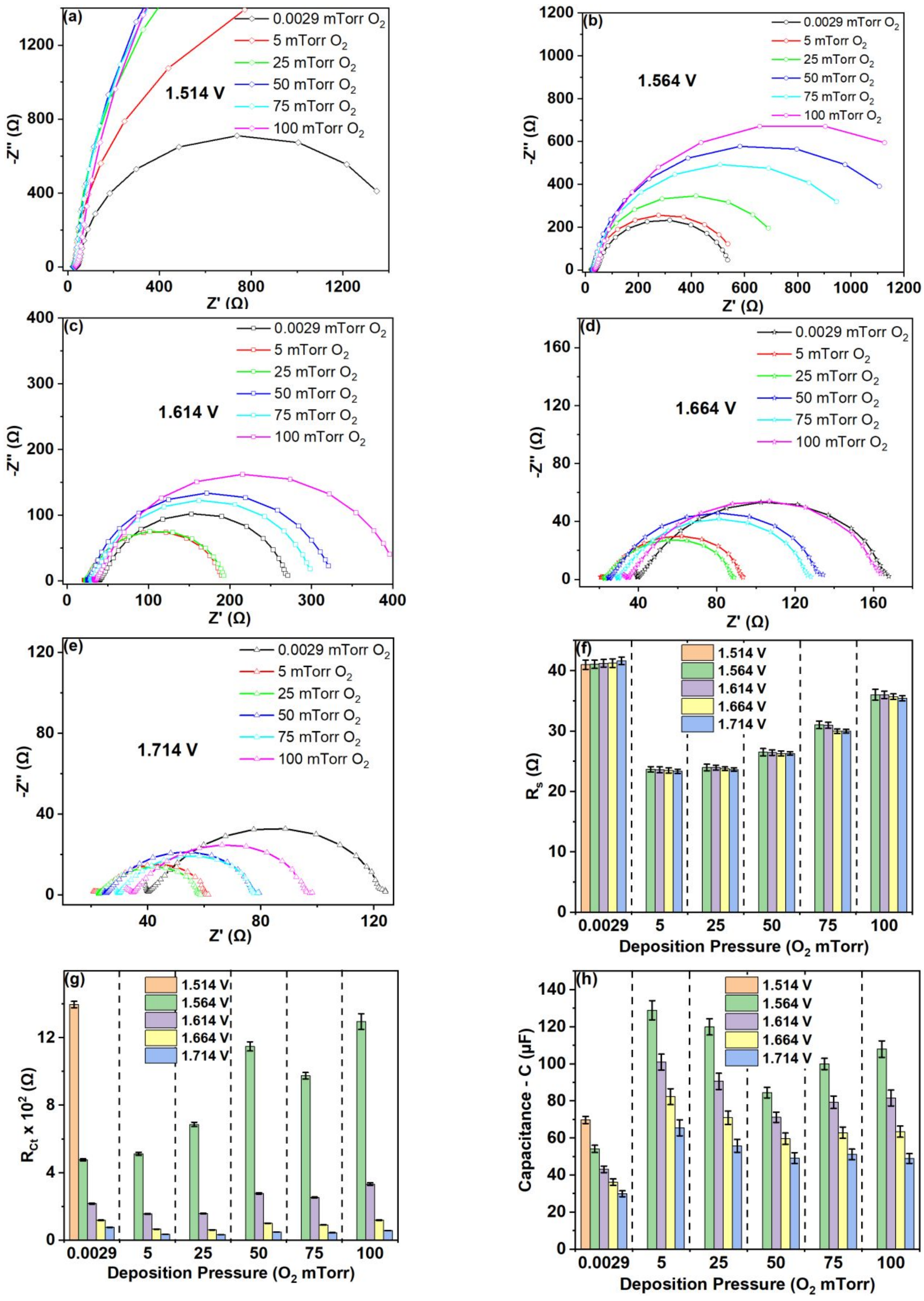

Figure S12. Nyquist plots of the various RuO_2 thin films at various potentials in 0.1 M KOH for (a) 1.514 V, (b) 1.564 V, (c) 1.614 V, (d) 1.664 V, (e) 1.714 V vs RHE, (f) Solution Resistance, (g) Charge Transfer Resistance, and (h) Capacitance at varying potentials.

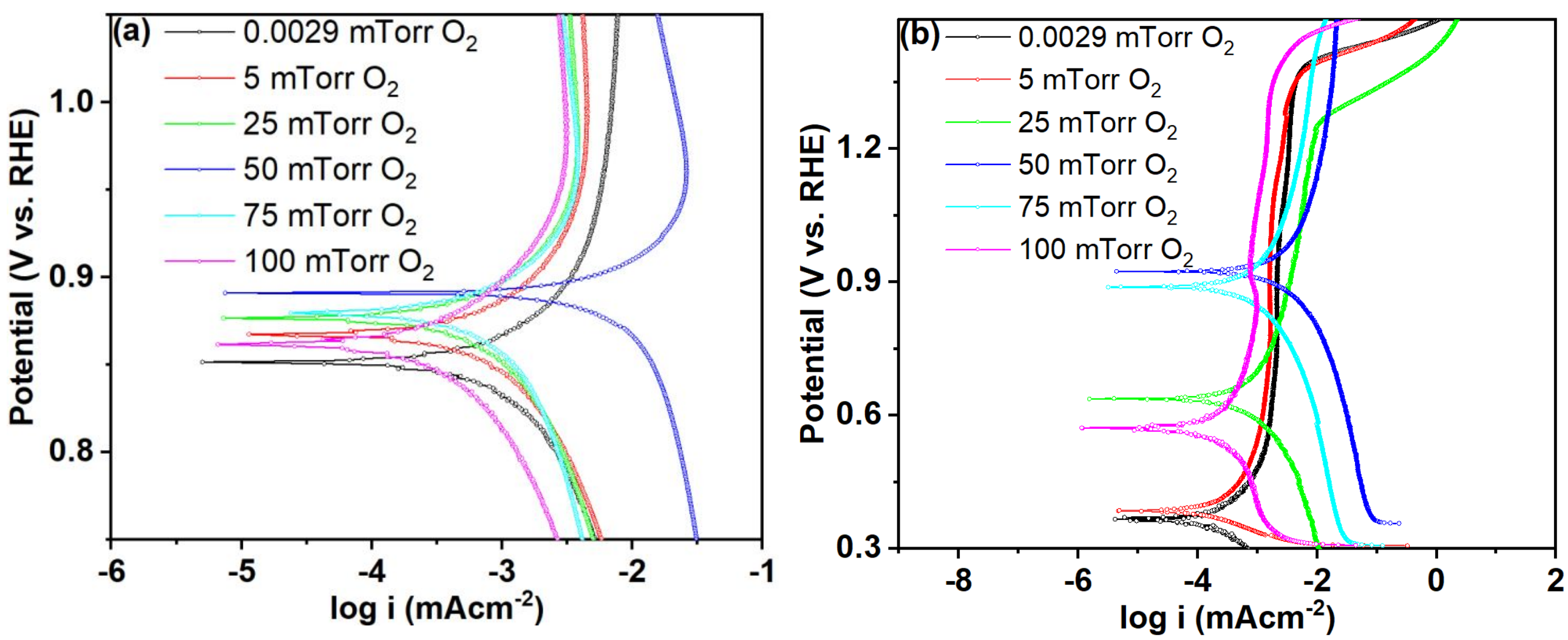

Figure S13. Corrosion analysis for the various RuO_2 films after 3 hrs. of CA measurements in (a) 0.1 M KOH and (b) 0.1 M $HClO_4$.

0.1 M KOH	E_{corr} (V)	β_a (V)	β_c (V)****	I_{corr} (mAcm^{-2})
0.0029	0.851	1.12766 ± 0.02051	-0.60725 ± 0.0042	-2.279
5	0.867	0.69187 ± 0.046487	-0.44289 ± 0.00151	-2.302
25	0.877	0.45445 ± 0.129992	-0.59071 ± 0.00404	-2.362
50	0.891	0.58508 ± 0.079046	-0.64442 ± 0.00173	-1.676
75	0.88	0.80688 ± 0.15976	-0.62587 ± 0.00353	-2.501
100	0.861	0.52715 ± 0.04452	-0.43555 ± 0.00215	-2.686

Table S6. Highlights of the corrosion analysis based on corrosion current and potential, and the anodic and cathodic Tafel slopes for 0.1 M KOH. '*' is used to denote possibility of higher variation.

0.1 M $HClO_4$	E_{corr} (V)	β_a (V)	β_c (V)	I_{corr} (mAcm^{-2})
0.0029	0.363	1.2494 ± 0.01249	-0.56012 ± 0.00476***	-2.917
5	0.39	1.07377 ± 0.0344	-0.50807 ± 0.02022***	-2.814
25	0.637	0.61859 ± 0.01982	-0.60135 ± 0.00297	-2.542
50	0.924	1.49973 ± 0.01555	-0.60233 ± 0.00247	-2.553
75	0.889	0.92135 ± 0.00454	-0.62434 ± 0.00326	-2.558
100	0.567	0.72114 ± 0.01354	-0.43769 ± 0.00667	-3.337

Table S7. Highlights of the corrosion analysis based on corrosion current and potential, and the anodic and cathodic Tafel slopes for 0.1 M $HClO_4$.

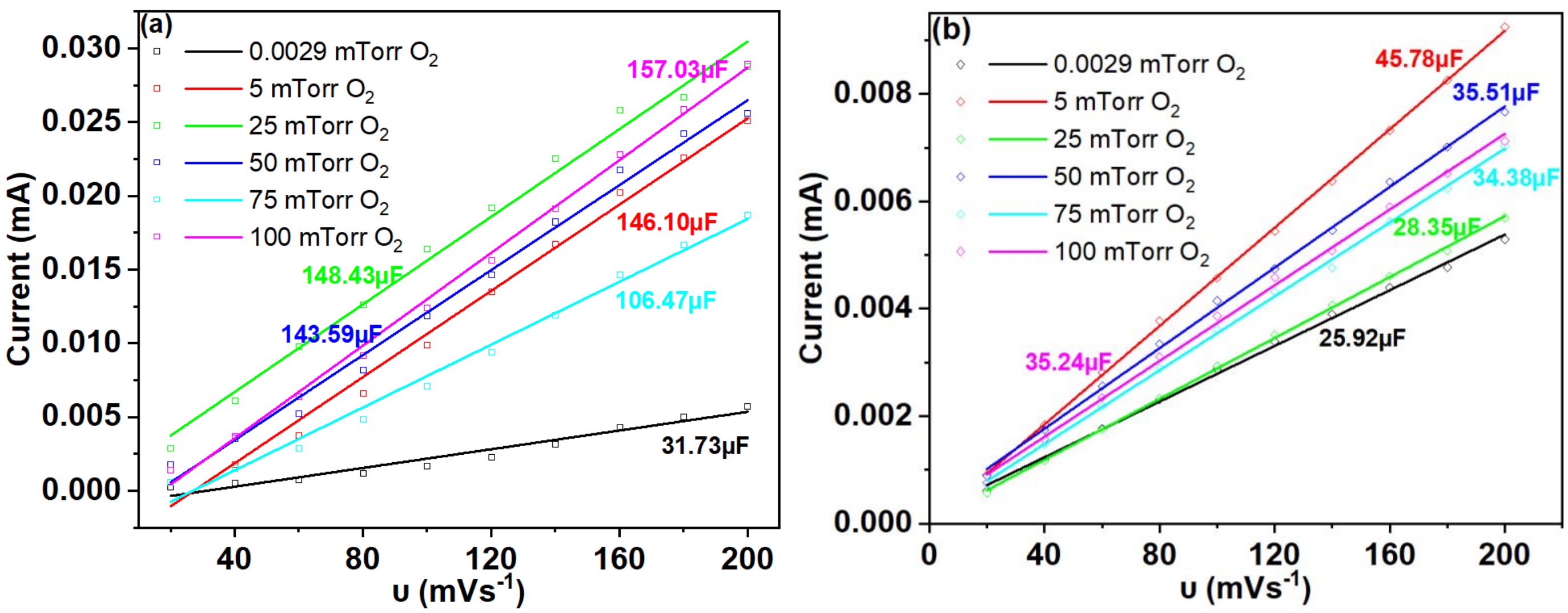

Figure S14. Electrical double layer capacitance (EDLC) as a function of scan rate in (a) 0.1 M KOH and (b) 0.1 M $HClO_4$.

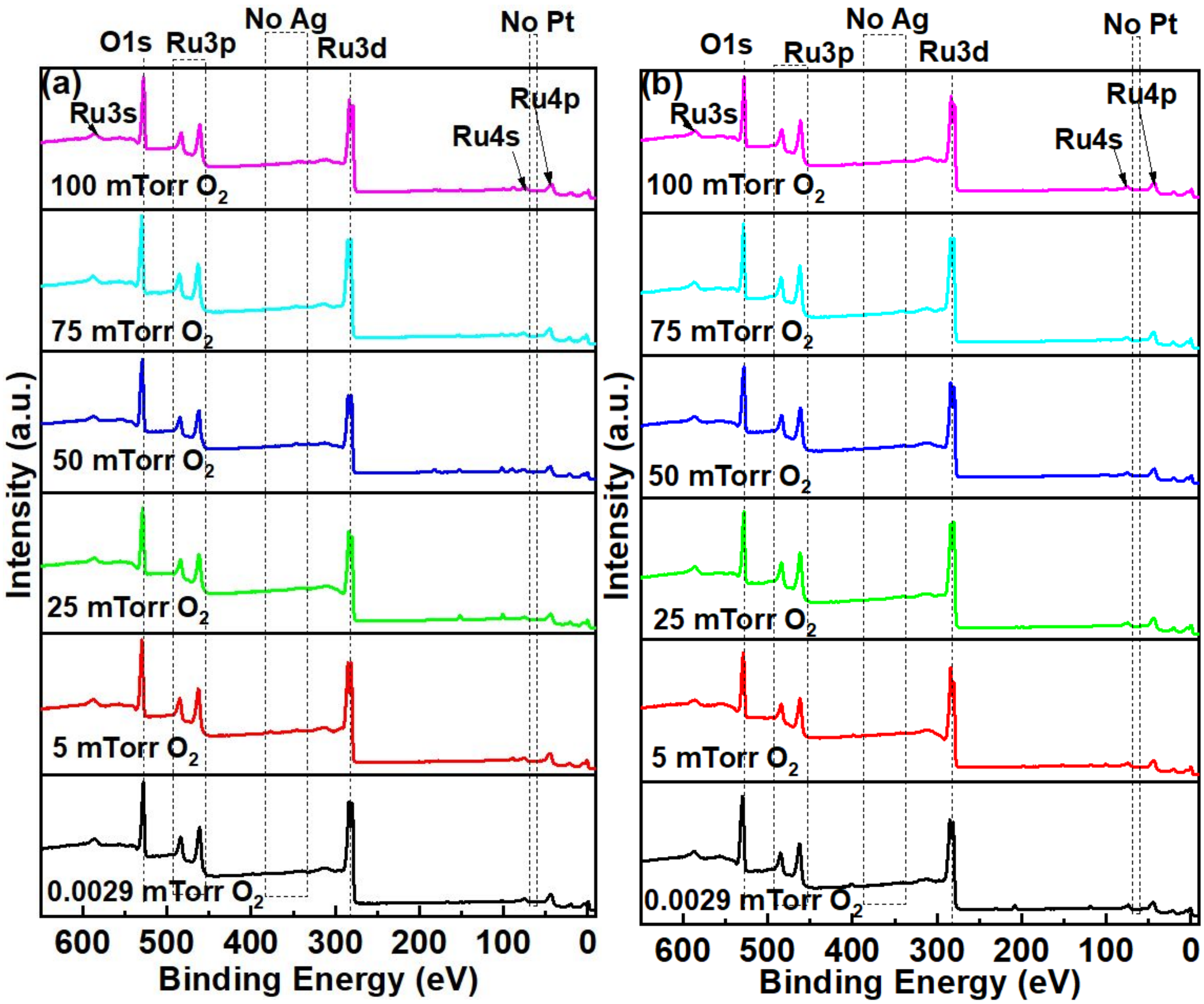

Figure S15. Post-chronoamperometry surface analysis for the various RuO_2 films showing chemical purity and absence of metal that may arise from electrolyte contamination such as Ag, Pt, Fe, Cu, Ni, Co, Mn, etc. for (a) 0.1 M KOH and (b) 0.1 M $HClO_4$.

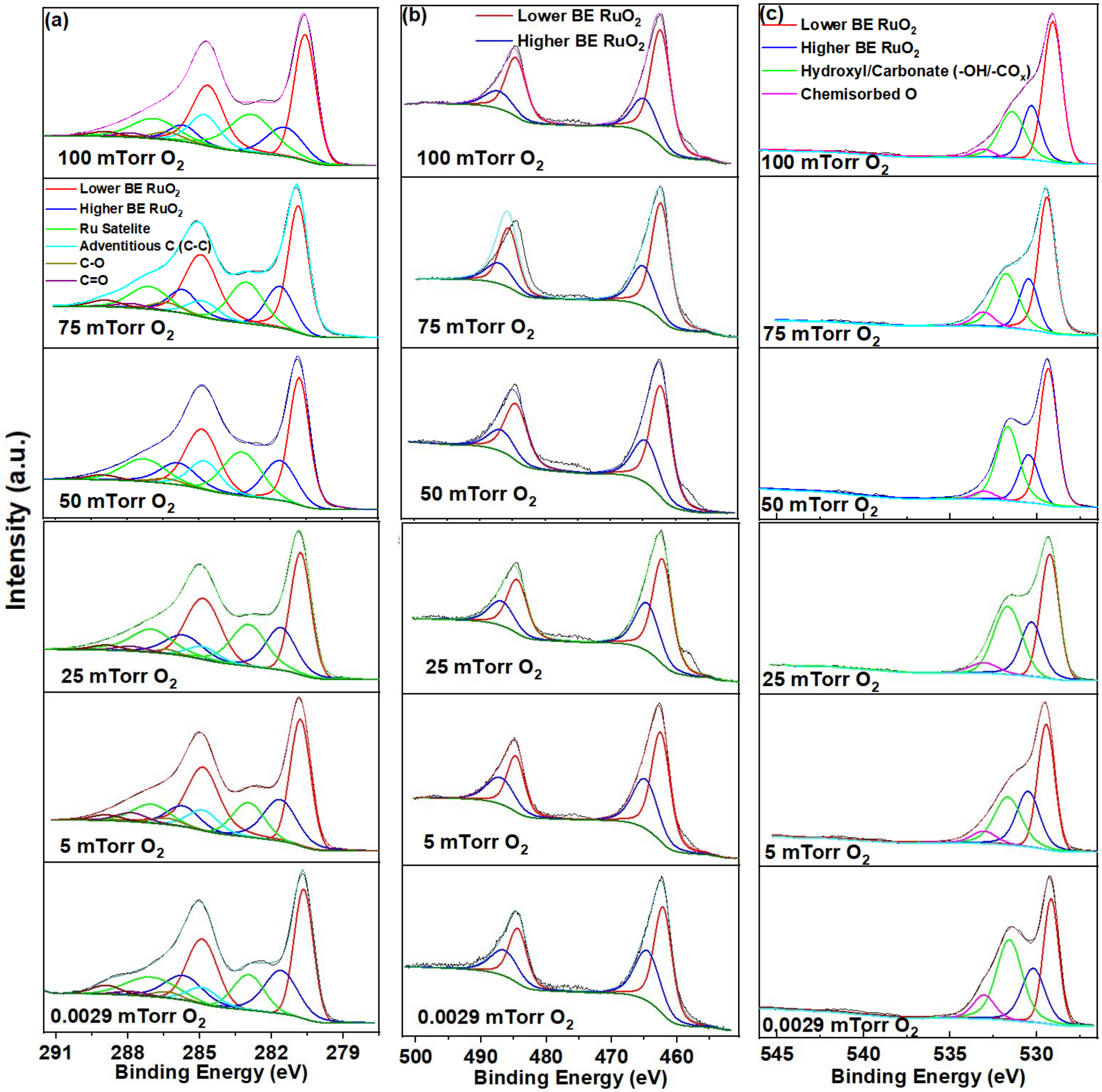

Figure S16. High-resolution XPS of the various samples after 12 hrs. of Chronoamperometry in 0.1 M KOH for (a) Ru 3d core level, (b) Ru 3p core level, and (c) O 1s core level.

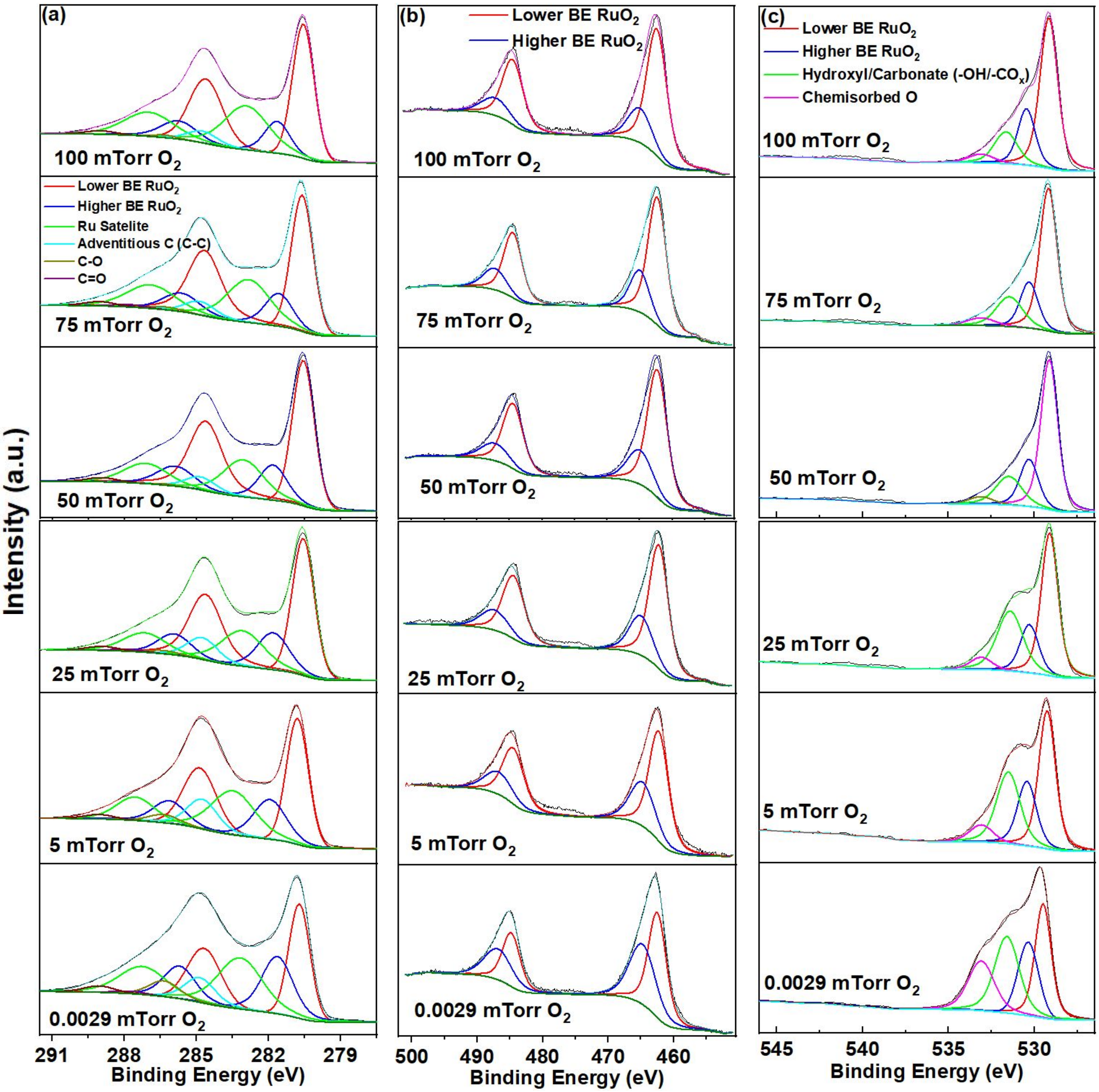

Figure S17. High-resolution XPS of the various samples after 12 hrs. of Chronoamperometry in 0.1 M $HClO_4$ for (a) Ru 3d core level, (b) Ru 3p core level, and (c) O 1s core level.

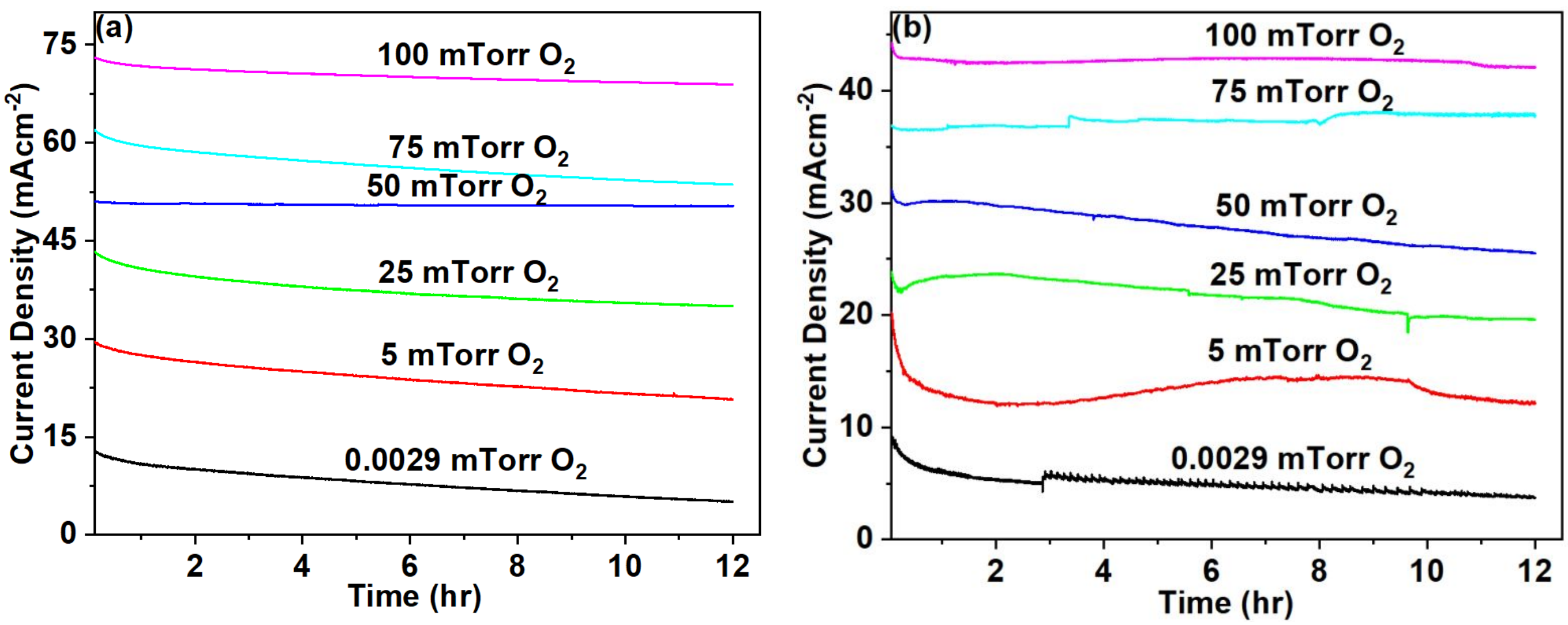

Figure S18. Chronoamperometric analysis of the various RuO_2 films performed at 1.9 V vs RHE over 12hrs and Ruthenium dissolution measured by ICP-MS in (a) 0.1 M KOH and (b) 0.1 M $HClO_4$.

Supporting Note S1 ICP-MS Measurement and Faradaic Efficiency (FE) of Dissolution

The amount of Ru metal dissolved in the electrolyte is normalized to geometric surface area; before that, the concentration ranged from 0.1426 to 8.2818 ppb in 0.1 M KOH and from 22.94 to 143 ppb in 0.1 M $HClO_4$. The measurements were performed once in each electrolyte, and since the values are low and similar to literature values, they are acceptable.

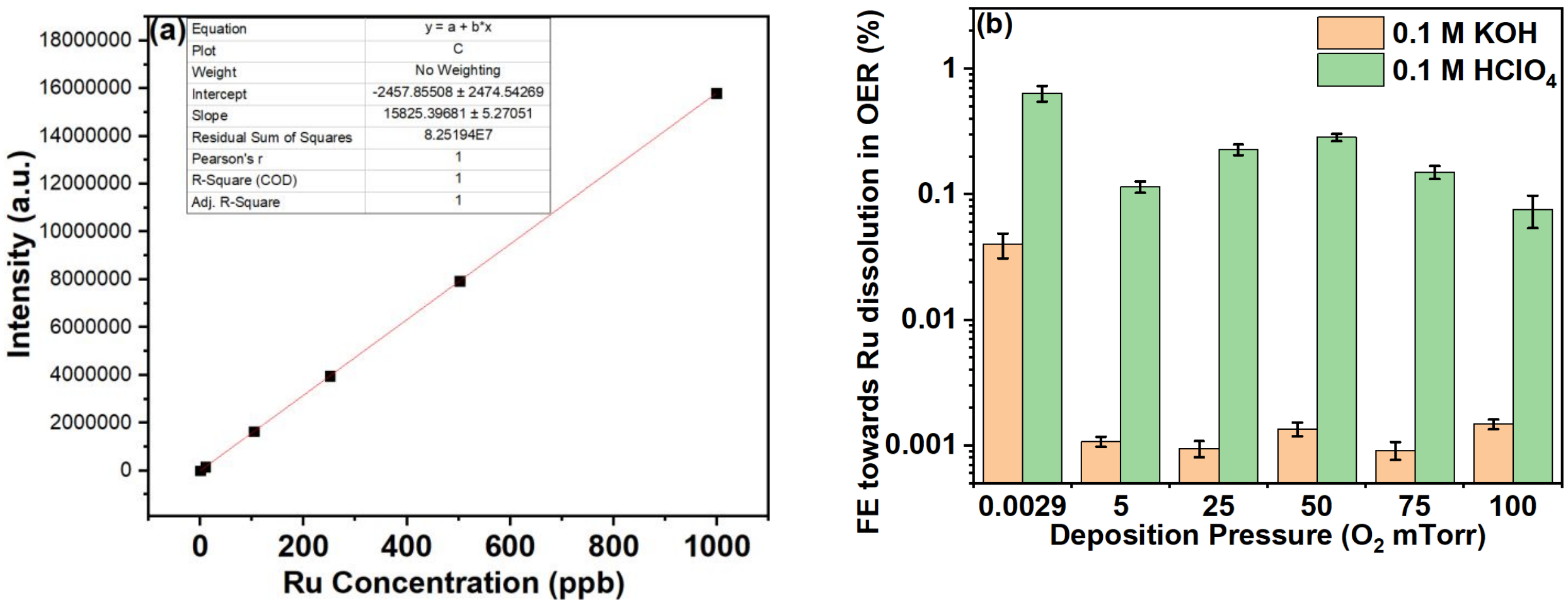

Figure S19. Calibration curve used in the ICP-MS to approximate the Ru metal concentration dissolved in the electrolyte of (a) 0.1 M KOH and 0.1 M $HClO_4$ and (b) Comparison of the Faradaic efficiency of dissolution for Ru-based catalysts in acidic and alkaline electrolytes

Calculation of Faradaic Efficiency towards dissolution

These Faradaic efficiency measurements are based on 12 hrs. of Chronoamperometry in both acidic and alkaline conditions at 1.9 V vs RHE. Faradaic efficiency (also Faradaic selectivity towards dissolution) is the ratio/percentage of electrical charge ($Q_{dissolution}$) that is involved in making the desired final chemical product during an electrochemical reaction. It compares the actual number of products derived (in this case dissolved Ru) to the theoretical maximum (Q_{total}) predicted by Faraday's law of electrolysis. In simpler terms, it measures the amount of Ru dissolved to produce an amount of current/electricity which could correspond to oxygen evolved. This process takes place in 4 basic steps as presented below (with reference to equation S1-S5).

1. Measurement of the integrated area under the chronoamperometry plot gives the total charge transferred over time (Q_{total}), as is seen in Figure S19 for all samples.

2. The total charge is related to the dissolution of Ru. The corrosion of RuO_2 and Ru is presented below.

$RuO_2 + 2H_2O \rightarrow RuO_{4(aq)} + 4H^+ + 4e^-$……………………………………………………………(S1)

$Ru + 8H_2O \rightarrow RuO_{4(aq)} + 8H^+ + 8e^-$…………………………………………………………… (S2)

3. Where 4 electrons are exchanged per mole of dissolution of RuO_2 and 8 electrons per mole of Ru dissolution. The charge of dissolution ($Q_{dissolution}$) is presented below.

$$Q_{dissolution} = \frac{m\ .\ F\ .\ z}{M} \text{ .... (S3)} \quad m_{RuO2} = \frac{MRuO2}{MRu}\ .\ m_{Ru} \text{......... (S4)}$$

4. Where m_{RuO2} is the mass of RuO_2 dissolved (as that is the electrode material), F is the Faraday Constant 96485.332 $Cmol^{-1}$, z is the number of electrons exchanged (4 in our case), and M is the molar mass of RuO_2. The mass of Ru must be converted into RuO_2 as presented above.

5. Eventually, the Faradaic efficiency in % is calculated as a ratio of the calculated dissolution charge (step 4) and the total charge derived from the chronoamperometry plot (step 1)

$$FE\ (\%) = \frac{Qdissolution}{Qtotal}\ x\ 100\% \text{.................................(S5)}$$

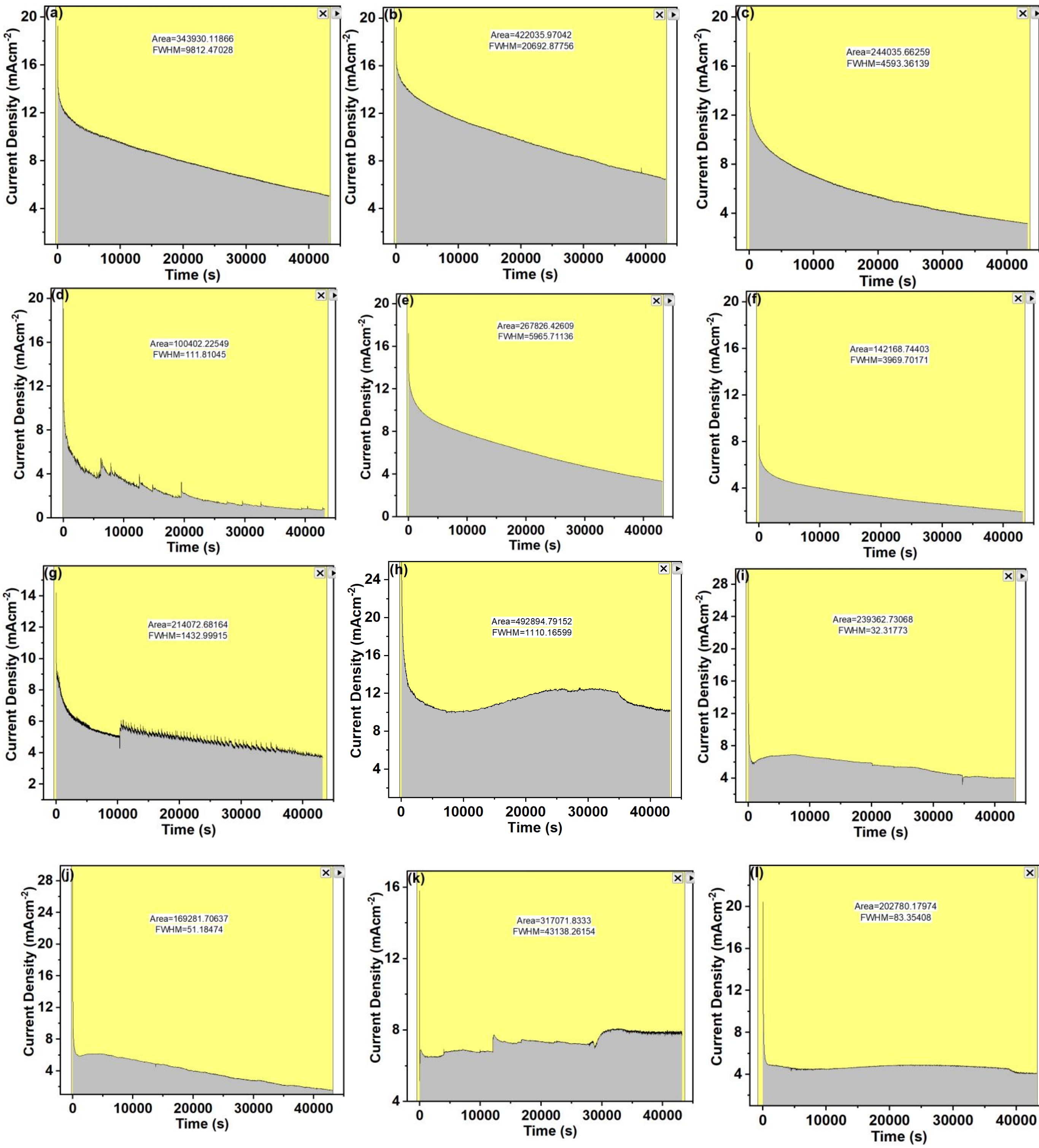

Figure S20. Chronoamperometry plot showing the amount of charge transferred in 12 hrs for (a) 0.0029 mTorr O_2, (b) 5 mTorr O_2, (c) 25 mTorr O_2, (d) 50 mTorr O_2, (e) 75 mTorr O_2, (f) 100 mTorr O_2 in 0.1 M KOH and, (g) 0.0029 mTorr, (h) 5 mTorr O_2, (i) 25 mTorr O_2, (j) 50 mTorr O_2, and (k) 75 mTorr O_2 (l) 100 mTorr O_2 in 0.1 M $HClO_4$.

Supporting Note S2 Calculation of Turnover Frequency (TOF)

The intrinsic site activity for RuO_2 (100) in alkaline and acidic conditions was calculated from the turnover frequency, which defines/quantifies the efficiency with which the electrocatalyst converts species into final products at a single active site per unit time. It explains how quickly the active site works independently of surface area or catalyst loading (with reference to equation S6-S9).

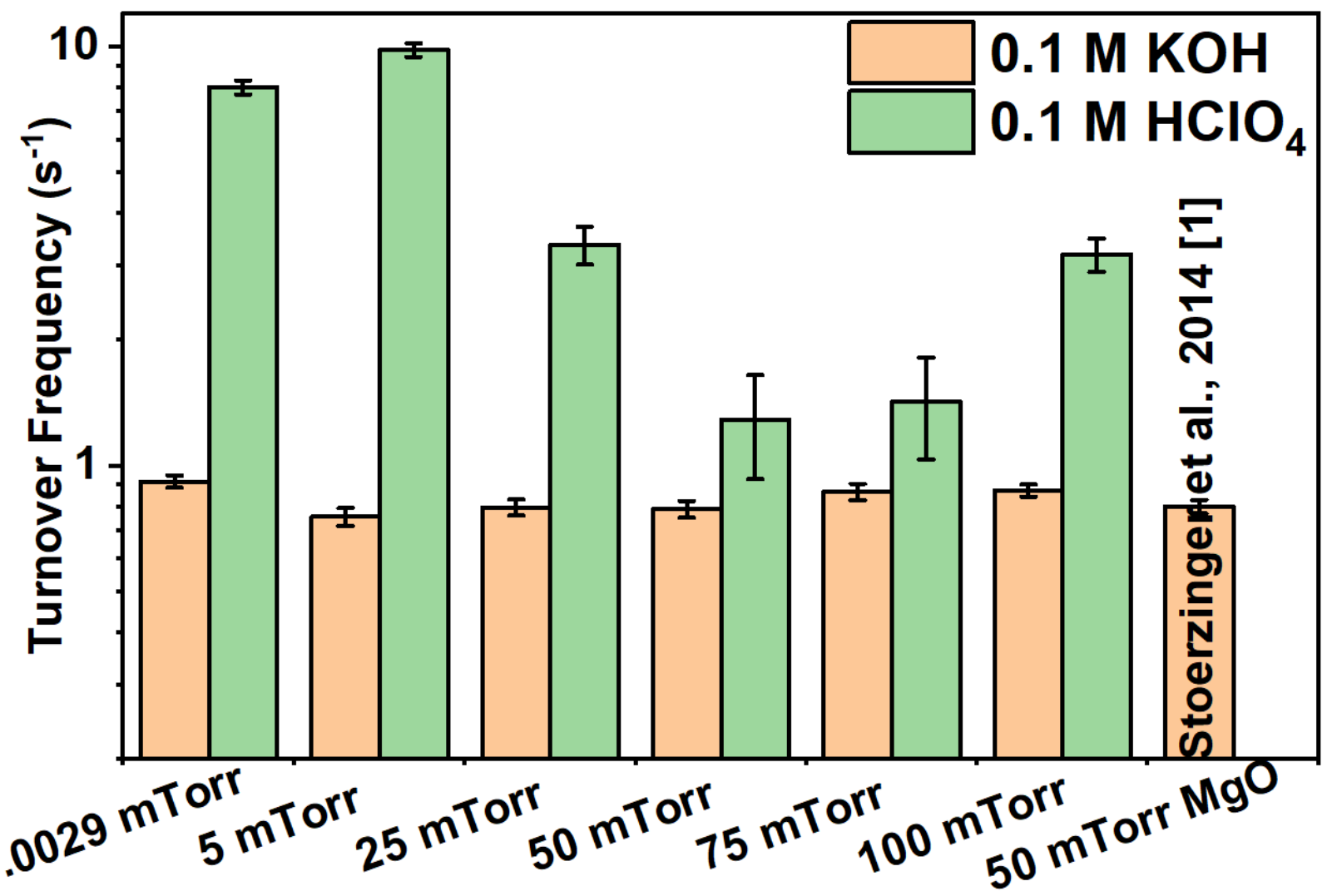

Figure S21. Comparison of the Turnover frequency for RuO_2 in acidic and alkaline electrolytes with data adapted from Stoerzinger et al., 2014 [1, 2]

Calculation of Turnover Frequency

$$\mathrm{TOF} = \frac{N_{product}}{N_{active\ sites} \times t} \quad \text{(S6)}$$

Where: $N_{product}$ = number of product molecules formed; $N_{active\ sites}$ = number of active sites (mol)
t = reaction time (s)

In electrochemistry;

$$\mathrm{TOF} = \frac{I}{n \times F \times N_{active\ sites}} \quad \text{(S7)}$$

Where: I = current (A); n = number of electrons per product mol (HER=2, OER=4); F = Faraday constant = 96485 $C.mol^{-1}$.g

$$N_{active\ sites} = \frac{Q_{redox}}{F \times n_{redox}} \quad \text{(S8)}$$

Where: Q_{redox} = integrated redox charge from CV; n_{redox} = electrons involved in redox (RuO_2=1)

$$Q_{redox} = \frac{\int I.\delta V}{\upsilon} \quad \text{(S9)}$$

Where: δV = Voltage range of integrated redox scan; υ = scan rate of CV (200 mVs^{-1})

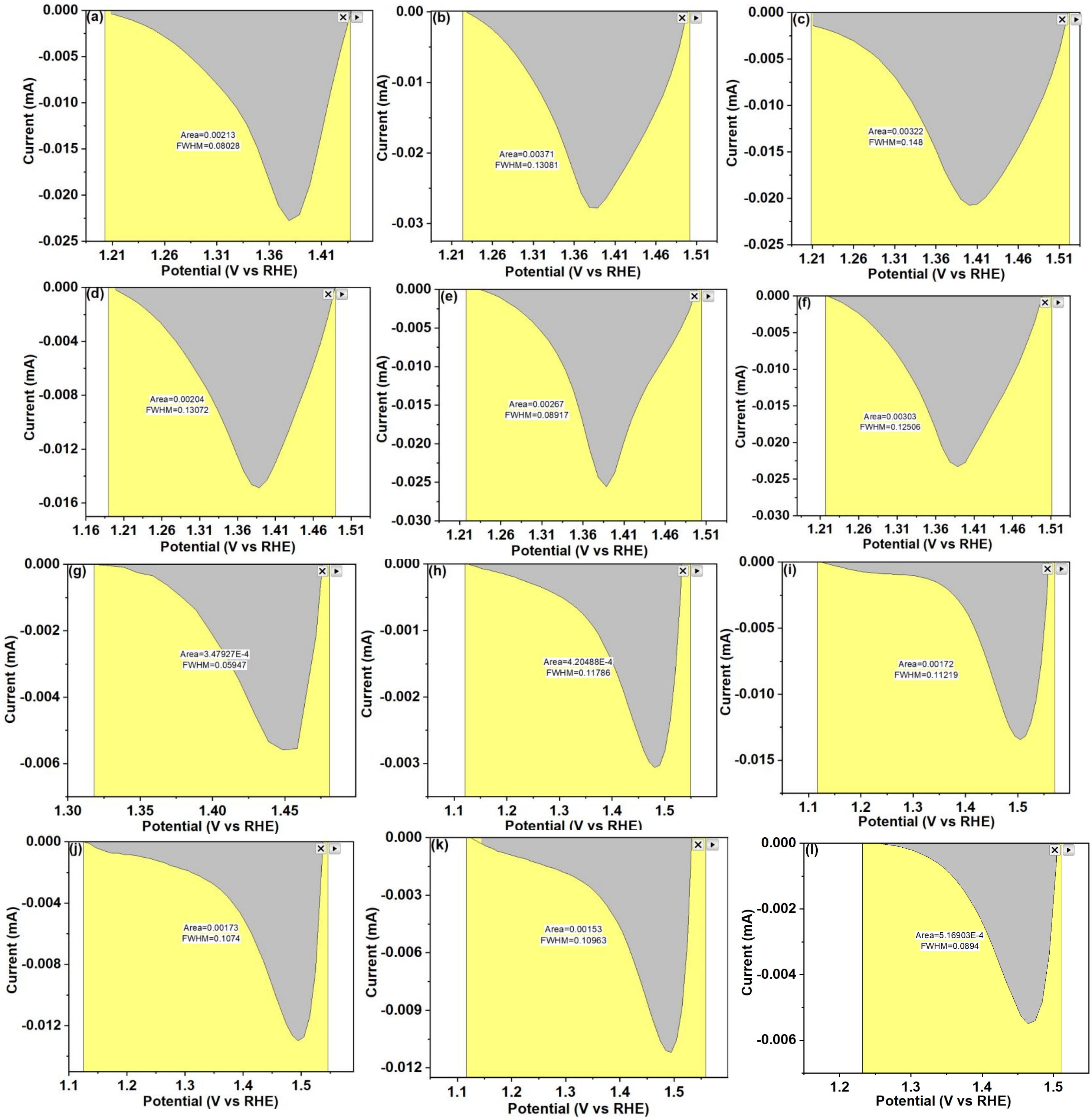

Figure S21. Integral of the O* in alkaline and OOH* in acid for Q_{redox} calculation in (a) 0.0029 mTorr O_2 (b) 5 mTorr O_2 (c) 25 mTorr O_2 (d) 50 mTorr O_2 (e) 75 mTorr O_2 (f) 100 mTorr O_2 in 0.1 M KOH and (g) 0.0029 mTorr (h) 5 mTorr O_2 (i) 25 mTorr O_2 (j) 50 mTorr O_2 (k) 75 mTorr O_2 (l) 100 mTorr O_2 in 0.1 M $HClO_4$.

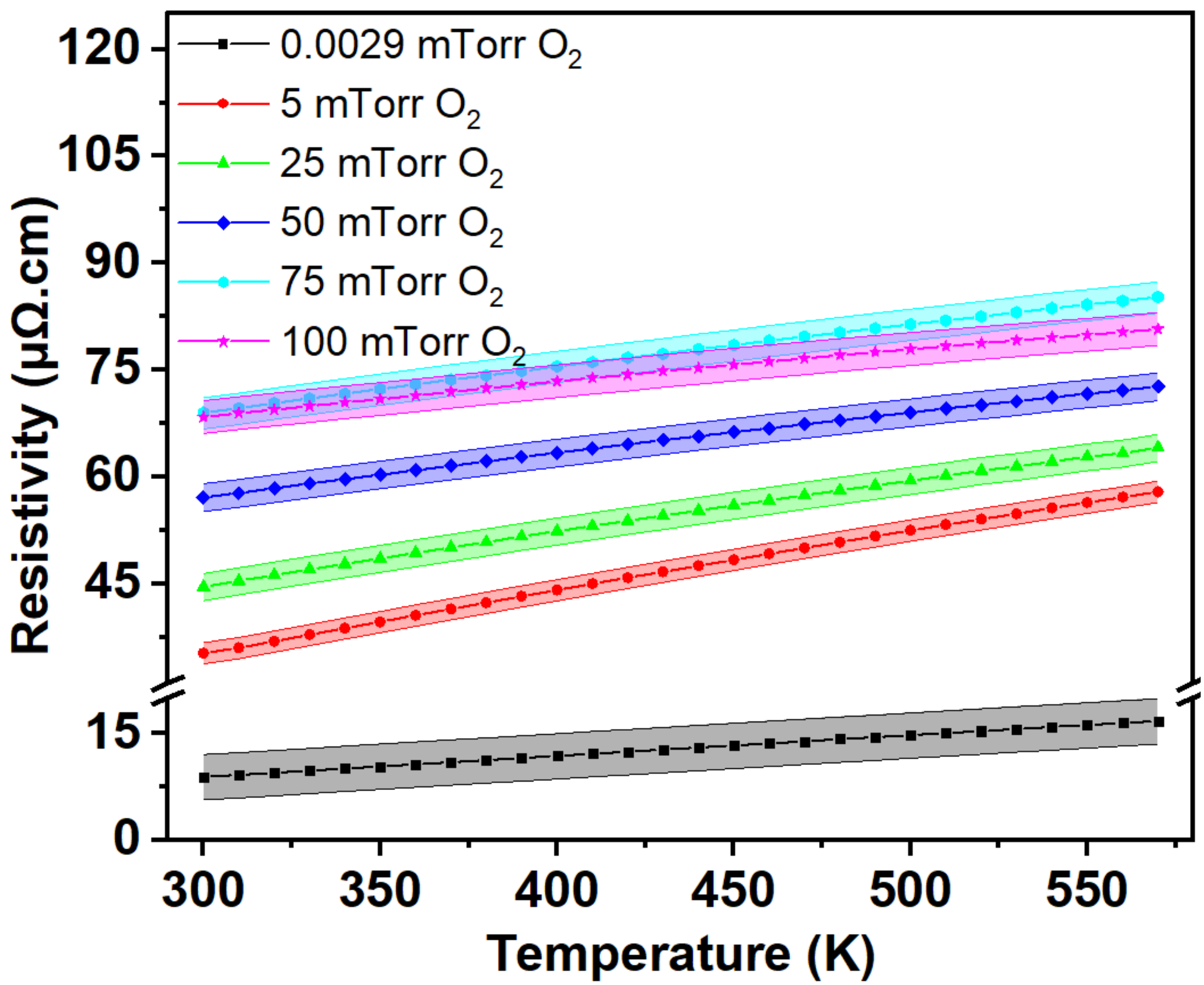

Figure S22. Four-probe resistivity as a function of temperature for the various RuO_2 films.

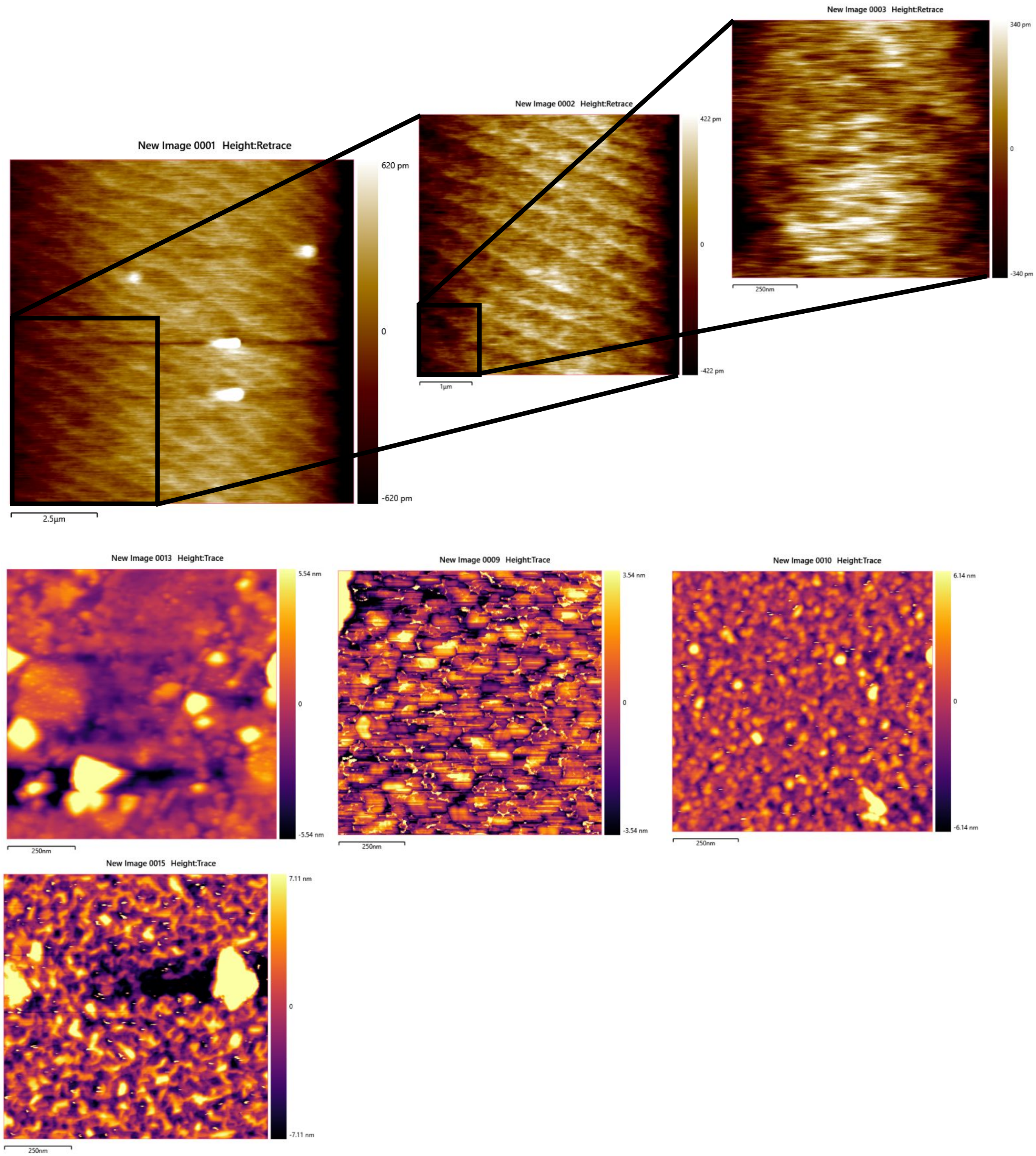

Figure S23. AFM images of (a-c) Al_2O_3 HNO_3 cleaned showing the characteristics atomically flat surface when observed at 10 µm x 10 µm, 5 µm x 5 µm, 1 µm x 1 µm. Deposited films at 1 µm x 1 µm for (d) 0.0029 mTorr O_2, (e) 5 mTorr O_2, (f) 25 mTorr O_2, (g) 50 mTorr O_2 samples with Root-Mean-Square (RMS) Roughness ranging between 3-6 nm.

REFERENCES

[1] K.A. Stoerzinger, L. Qiao, M.D. Biegalski, Y. Shao-Horn, Orientation-dependent oxygen evolution activities of rutile IrO2 and RuO2, The journal of physical chemistry letters 5(10) (2014) 1636-1641.

[2] E.A. Paoli, F. Masini, R. Frydendal, D. Deiana, C. Schlaup, M. Malizia, T.W. Hansen, S. Horch, I.E. Stephens, I. Chorkendorff, Oxygen evolution on well-characterized mass-selected Ru and RuO 2 nanoparticles, Chemical science 6(1) (2015) 190-196.
